# *Capparis cartilaginea* decne (capparaceae): isolation of flavonoids by high-speed countercurrent chromatography and their anti-inflammatory evaluation

**DOI:** 10.3389/fphar.2023.1285243

**Published:** 2023-10-19

**Authors:** Bashaer Alsharif, Nadhim Hante, Bruna Govoni, Hugo Verli, Wirginia Kukula-Koch, María Jose Santos-Martinez, Fabio Boylan

**Affiliations:** ^1^ School of Pharmacy and Pharmaceutical Sciences, Trinity Biomedical Sciences Institute, Trinity College Dublin, Dublin, Ireland; ^2^ Department of Pharmacognosy, Faculty of Pharmacy, Umm Al-Qura University, Makkah, Saudi Arabia; ^3^ Faculty of Pharmacy, University of Kufa, Al-Najaf, Iraq; ^4^ Center of Biotechnology, Federal University of Rio Grande do Sul, Porto Alegre, Brazil; ^5^ Department of Pharmacognosy with Medicinal Plants Garden, Medical University of Lublin, Lublin, Poland; ^6^ School of Medicine, Trinity College Dublin, Dublin, Ireland

**Keywords:** flavonoids, capparis, HSCCC, anti-inflammatory, LC-MS, virtual screening

## Abstract

**Introduction:** *Capparis cartilaginea* Decne. (CC) originates from the dry regions of Asia and the Mediterranean basin. In traditional medicine, tea of CC leaves is commonly used to treat inflammatory conditions such as rheumatism, arthritis, and gout. Due to the limited studies on the phytochemistry and biological activity of CC compared to other members of the Capparaceae family, this work aims to: 1) Identify the chemical composition of CC extract and 2) Investigate the potential anti-inflammatory effect of CC extract, tea and the isolated compounds.

**Methods:** To guarantee aim 1, high-speed countercurrent chromatography (HSCC) method; Nuclear Magnetic Resonance (NMR) and High-Performance Liquid Chromatography coupled to Electrospray Ionisation and Quadrupole Time-of-Flight Mass Spectrometry (HPLC-ESIQTOF-MS/MS) were employed for this purpose. To guarantee aim 2, we studied the effect of the isolated flavonoids on matrix metalloproteinases (MMPs) −9 and −2 in murine macrophages. Molecular docking was initially performed to assess the binding affinity of the isolated flavonoids to the active site of MMP-9.

**Results and discussion:** *In silico* model was a powerful tool to predict the compounds that could strongly bind and inhibit MMPs. CC extract and tea have shown to possess a significant antioxidant and anti-inflammatory effect, which can partially explain their traditional medicinal use.

## 1 Introduction

Inflammation is a complex physiological response of the body to harmful stimuli, such as pathogens, damaged cells, or irritants. It is a highly regulated process coordinated by the immune system, involving a series of complex interactions between various immune cells, signaling molecules, and tissues. While inflammation is essential for eliminating the cause of injury or infection and promoting tissue repair, its dysregulation can lead to a wide range of chronic diseases and disorders (Chen et al., 2018).

Matrix metalloproteinases (MMPs) are a large family of proteolytic enzymes that break down proteins and proteoglycan elements within the extracellular matrix (ECM) (Chen et al., 2018). They are involved in physiological processes associated with homeostasis regulation, host defense, and tissue repair. However, they also are known to play a key role in the development and progression of inflammatory diseases. MMP-2 and MMP-9 are referred to as gelatinases for their ability to digest gelatin. MMP-2 (72 kDa) is constitutively expressed, whereas MMP-9 is an inducible enzyme that shows limited expression in healthy tissues but becomes notably activated in a number of pathological conditions. In fact, disruption of MMPs expression and/or their function can cause prolonged inflammatory response, leading to chronic inflammation (Köhrmann et al., 2009). MMP-2 is involved in angiogenesis, tissue repair, regulation of vascularization and inflammatory response (Cui et al., 2017). MMP-9 (92 kDa), is produced by a wide numbers of cells and by activated macrophages (Galis et al., 1995). Its primary function involves degrading various components of the extracellular matrix, such as gelatin, collagen, and laminin, which allows immune cells to migrate through tissues to reach the site of inflammation more effectively (Nishikaku et al., 2009). MMP-9 expression is upregulated in inflammatory, malignant, and degenerative disorders (Loftus et al., 2000) suggesting that inhibition of MMP-9 activity could potentially hold therapeutic benefits.

Flavonoids are a class of naturally occurring phenolic compounds and well-known anti-inflammatory agents (Loftus et al., 2000). They can exert their anti-inflammatory effect by inhibiting MMPs or preventing their activation (Lim et al., 2019). For instance, compounds such as quercetin, kaempferol, and hyperoside, and catechins have previously demonstrated to inhibit MMP-2 and -9 gelatinase activity (Sartor et al., 2002; Lim et al., 2019). Furthermore, several flavonoids such as quercetin and nobiletin have been reported to downregulate MMP-1 expression in human vascular endothelial cells, human synovial fibroblasts, and UVA-irradiated human dermal fibroblasts (HDFs) (Song et al., 2001; Lin et al., 2003).

*Capparis* are known to be rich sources of phenolic compounds, mainly flavonoids (Grimalt et al., 2022). *Capparis cartilaginea* Decne (CC) is a member of the genus Capparis, which is the largest genus in the family Capparaceae (Maurya et al., 2023). Native to arid regions, particularly in north and east Africa and in the Middle East, this plant has adapted to adverse climatic conditions without showing signs of stress (Chedraoui et al., 2017). CC is known for its distinctive features, such as leathery leaves, white or pink flowers, and elongated fruit capsules (Lansky et al., 2013). Beyond its aesthetic appeal, the plant has been used for centuries for its medicinal value. In traditional medicine CC is used as tea for the treatment of arthritis and rheumatism, earache, headache, healing of bruises, snakebite, and pain associated to childbirth (Hamed et al., 2007; Alzweiri et al., 2011). In Africa, CC roots are used for dermatitis and skin ulcers, while tea from the leaves is used for dyspepsia. Furthermore, leaves, twigs, and stems of CC are also chewed for relieving colic (Rivera et al., 2003).

Because the wide traditional uses of CC suggest possible anti-inflammatory and analgesic effects, and due to the limited number of chemical and pharmacological studies that have been conducted on CC to demonstrate their potential value in this regard, the aims of this study were to isolate the primary flavonoids from CC extract using HSCCC and to investigate the impact of the isolated flavonoids on MMP-9. Due to the widespread folk use of CC in traditional medicine, we hypothesize that this plant and the main compounds in its tea in fact have an anti-inflammatory effect. To test this hypothesis, an *in silico* evaluation was carried out through molecular docking experiments, as well as *in vitro* using lipopolysaccharide (LPS)-activated murine macrophages (RAW264.7) with the isolated flavonoids from the tea produced with CC.

## 2 Materials and methods

### 2.1 Chemicals

All solvents used for the extraction and chromatographic procedures were of HPLC-grade quality and purchased from the Hazardous. Materials Facility (HMF) in Trinity College Dublin (Dublin, Ireland). Deionized water was obtained from a Millipore™ water deionizing system. Sephadex^®^ LH-20 was purchased from Sigma-Aldrich (Arklow, Ireland). The solvents employed for Nuclear Magnetic Resonance (NMR) analysis included Methanol-D4 (≥99.8% purity) and Dimethyl Sulfoxide-D6 (DMSO, 99.9% purity) from Sigma-Aldrich (Arklow, Ireland). Lipopolysaccharide (LPS) from *Escherichia coli* 0111: B4 (L4391), fetal bovine serum (FBS, F7524), and Dulbecco’s modified Eagle’s medium (DMEM, D0822) were also sourced from Sigma-Aldrich (Arklow, Ireland). The Cell Counting Kit-8 (CCK-8) was obtained from Dojindo, and dexamethasone was acquired from Sigma-Aldrich (CAS No. 50–02–2).

### 2.2 Plant collection

Samples of CC fresh leaves were collected from Al-Taif region, Saudi Arabia, in June 2019. The plant was identified by Professor Ammar Bader, and voucher specimens (SA-UK 2019-2) were deposited in the pharmacognosy lab’s herbarium at Umm Al-Qura University. The collected leaves were air-dried to obtain a stable weight and finely ground.

### 2.3 Preparation of CC extract

CC extracts were prepared by infusing 500 g of the plant leaves powder in 2.5 L boiling water. After 15 min, the brew was filtered using muslin cloth and Whatman no.1 filter paper. Then, the tea was partitioned with ethyl acetate and butanol (100 mL) ten times and concentrated with a rotary evaporator (ratio of 10:1, infusion to organic solvent). The organic extracts obtained (5.2 g of ethyl acetate extract and 9.8 g butanol extract) were combined and suspended in the solvent system used for further analysis (referred throughout the paper as extract).

### 2.4 HPLC-ESI-QTOF-MS/MS analysis

A High-Performance Liquid Chromatography coupled to Electrospray Ionisation and Quadrupole Time-of-Flight Mass Spectrometry (HPLC-ESI-QTOF-MS/MS) apparatus produced by Agilent Technologies (Santa Clara, CA, US) was employed for HPLC-MS analysis in the Department of Pharmacognosy with Medicinal Plants Garden of the Medical University of Lublin, Poland. The instrument was composed of HPLC chromatograph (1260 Series) equipped with a Zorbax Stable Bond RP-18 Column (150 mm × 2.1 mm, dp = 3.5 µm), a degasser (G1322A) with a binary pump (G1312C), an autosampler (G1329B), a photodiode array detector—DAD (G1315D), and a mass spectrometer (G6530B) with a quadrupole and a time-of-flight analysers. Agilent Mass Hunter Workstation Software (version B.08.00) was used to acquire the MS spectra and to handle the data. The separation was achieved in the following gradient of eluent A (water) and eluent B (acetonitrile), both with addition of 0.1% formic acid: Rt (retention time) 0 min–1% of eluent B, Rt 5 min 25% of eluent B, Rt 30 min 65% of eluent B, Rt 32–33 min 95% of eluent B, Rt 34 min 1% of eluent B. In the acquired method the stop time was set at 45 min, the temperature at 25°C, the flow rate at 0.2 mL/min, the post time at 2 min and the injection volume at 10 µL. The mass spectra were recorded within the *m/z* range of 100–1200 Da. The gas and sheath gas temperatures were set at 275 and 325^◦^C, the gas flows at 12 L/min, the nebulizer pressure at 35 psig, the fragmentation energy at 110 V, and the capillary voltage at 3000 V. The MS/MS fragmentation pattern was recorded at 10 and 20 V for two the most intensive signals in each scan. After the collection of 1 spectrum, the selected *m/z* value was excluded for the following 0.3 min from the analysis to provide fragmentation data of other less intense signals. The Mass Hunter Workstation Software (Agilent Technologies, Santa Clara, CA, USA, version B.10.00) enabled both the conduction of analyses and the management of the obtained spectral data.

### 2.5 High speed counter current chromatography (HSCCC) fractionation of CC extract

#### 2.5.1 Equipment

HSCCC was carried out using IntroPrepTM (Quattro) (AECS-QuikPrep, Cornwall, UK). The apparatus is operated by centrifugal force with a rotation speed of 865 rpm. The column consists of Polytetrafluoroethylene PTFE tubing (tube diameter 2.0 mm, volume 136 mL) wrapped around a bobbin. The manual sample loop is 6 mL in size. Separations were performed at room temperature.

#### 2.5.2 Choice of the solvent system for HSCCC fractionations

The choice of solvent system was decided after comparing four solvent systems containing-hexane, ethyl acetate, methanol, and water H:E:M:W (1:6:1:6) (v/v/v/v); hexane, ethyl acetate, butanol, methanol, and water H:E:B:M:W (1:6:0.5:1:6) (v/v/v/v); butanol, ethyl acetate, and water E:B:W (4:1:5 and 2:3:5) (v/v/v/v). Approximately 2 mg of CC extract was dissolved in separate test tubes containing one of the tested solvent systems. Then the test tubes were shaken and the sample was allowed to partition between the two liquid phases. Equal aliquots of each phase were spotted side by side on silica gel TLC plates developed with the solvent system ethyl acetate: formic acid: acetic acid: water (10:2.6:2.6:1). The plates in the natural products reagent (NP/PEG) were visualized under UV light (254 and 365 nm). The solvent system used for this study was selected according to the distribution of the compounds in both the upper and lower phases of the biphasic solvent system. The system that provided the best distribution of compounds was E:B:W (2:3:5 v/v/v/v).

#### 2.5.3 Preparation of the solvent system

The selected solvent system was thoroughly equilibrated in a separatory funnel at room temperature. The two phases were separated shortly before use and degassed by sonication for 15 min. The sample solutions were prepared by dissolving the sample (1.5 g of CC extract) in the solvent mixture of the aqueous lower phase and organic upper phase (1:1 v/v) of the solvent system used for HSCCC separation.

#### 2.5.4 HSCCC separation procedure

The separation was carried out in the “head to tail” elution mode in which the upper organic layer acts as the stationary phase and the aqueous layer as the mobile phase. Afterwards, the extrusion mode was settled in which the upper organic layer acts as mobile phase and aqueous layer as stationary phase. The column was first entirely filled with the stationary phase, and then the mobile phase was pumped into the column at a flow rate of 2.0 mL/min, while the apparatus was rotated at 860 rpm, at a constant temperature of 25°C. The sample solution (1.5 g of CC extract in a 5 mL biphasic system) was injected into the injection valve after the system reached hydrodynamic equilibrium. The stationary phase retention Sf for the solvent system in each fractionation was around 84%–87%. Fractions were collected every 2 min for both the elution and the extrusion mode in 10 mL test tubes resulting in 150 fractions, each containing 4 mL eluent.

#### 2.5.5 Purification of isolated compounds

After successive cycles of HSCCC, the collected fractions were grouped into seven fractions according to the similarity of elution pattern and retention factor (Rf) observed in the TLC plates. Two semi-purified fractions (Loftus et al., 2000; Cui et al., 2017) were submitted to size exclusion Chromatography using a column Sephadex LH-20 obtained from Sigma-Aldrich (Arklow, Ireland), eluted with methanol at an average flow rate of 0.5–1 mL/min.

#### 2.5.6 NMR identification

Structural elucidation of the isolated molecules was performed using a Bruker Avance 400 instrument, at 400 MHz for proton (^1^HNMR) magnetic resonance and 100 MHz for carbon magnetic resonance (^13^C NMR). Spectral analysis was performed using the Mestrenova software. Chemical shifts are reported as (ppm) values, and the coupling constants are given in Hz. Two-dimensional measurements (H-H COSY, HMBC, HMQC) were obtained on the same instrument with the usual pulse sequences.

### 2.6 Antioxidant assays

#### 2.6.1 Determination of the total phenol content

The total phenolic content of CC extract and tea was determined using the Folin Ciocalteau assay according to the method described by Gursoy *et al.* with slight modifications (Gursoy et al., 2009). Gallic acid (GAE) was used as a standard and had the calibration curve plotted. The solutions of gallic acid were prepared in methanol at final concentrations of and 12.5, 25, 50, 100, 200, 400 μg/mL. In this assay, 4 µL o f the samples (400 μg/mL) were added to 180 µL o f distilled water and 4 µL of Folin Ciocalteau reagent. The sample was prepared in triplicate using 96 well plate. After adding the reagent, the plate was shaken vigorously and after 3 min, 12 µL of aqueous sodium carbonate (2%) solution w as added. The obtained mixture was kept at 40°C for 30 min and the absorbance values of the samples were measured at 760 nm. The measurement was recorded using BioTek Synergy H1 plate reader. The phenolic content of the samples was determined according to the gallic acid standard curve and the results were expressed as µg of gallic acid equivalents per mg of extract.

#### 2.6.2 Determination of the total flavonoids

The determination of the flavonoid equivalent of CC extract and tea was performed according to the method described by Gursoy *et al.* (Gursoy et al., 2009). Quercetin was used as a standard, and a calibration curve was plotted using solutions of quercetin prepared in methanol with final concentrations of 12.5, 25, 50, 100, 200, and 400 μg/mL. A blank solution consisted of 100 µL of extract solution and 100 µL of methanol without aluminum chloride (AlCl₃). In this assay, 100 µL of the extracts (400 μg/mL) were added to 100 µL of 2% AlCl₃ in methanol. The sample was prepared in four replicates using a 96-well plate. The absorbance reading was measured at 415 nm after 10 min. The flavonoid content of the samples was determined according to the quercetin standard curve, and the results were expressed as µg of quercetin equivalents per mg of extract. The same plate reader described before was used in this assay.

#### 2.6.3 ABTS radical scavenging activity

Radical scavenging activity of CC tea and extract against stable ABTS (2,2′-azino-bis(3-ethylbenzothiazoline-6-sulfonic acid) was carried out as previously described (Li et al., 2020). Ethanol was used to acquire an absorbance of 0.7 ± 0.02 at 734 nm for subsequent experiments. Then, 50 μL of the sample solution with different concentrations (12.5, 25, 50, 100, 200, and 400 μg/mL) was added to 150 μL of the diluted working solution. After vigorous shaking, the mixed solution was kept in the dark for 30 min. Afterwards, the absorbance of 200 μL of the mixed solution was measured at 734 nm. Ethanol was used as negative control in this study, and ascorbic acid was used as a positive control. The ABTS scavenging activity was calculated using the following equation:

$$\text{AA}\%=\left[\left(A_{\text{sample}}\;–\;A_{\text{negative}}\right)/A_{\text{negative}}\;\right]\times 100$$ (1)

A _blank_ and A _sample_ represent the absorbance of control and sample, respectively. The same plate reader described before was used in this assay.

#### 2.6.4 DPPH free radical scavenging activity assay

The radical scavenging activity of CC tea and extract against stable DPPH (l,l-diphenyl-2-picrylhydrazyl, Sigma-Aldrich) was determined spectrophotometrically according to a method adapted from Mensor *et al.* (Mensor et al., 2001). 100 μL of DPPH (0.2 mM) was added to 100 μL of samples with different concentrations (12.5, 25, 50, 100, 200, and 400 μg/mL), and the mixtures were placed in a dark room. The absorbance of the mixed solution was measured at 517 nm after a 35-min reaction. Ethanol was used as a negative control in this study, and ascorbic acid was used as a positive control. Eq. 1 was also utilized to calculate the activity of DPPH radical scavenging in this study. The same plate reader described before was used in this assay.

### 2.7 In *silico* assays

#### 2.7.1 Molecular docking

The *in silico* tests were carried out in order to gauge the potential interaction between the extracted flavonoids and human MMP-9, an enzyme that participates in several inflammatory processes and that has been previously described as potentially inhibited by flavonoids (Hilliard et al., 2020; Kubina et al., 2021). In order to evaluate the binding between the MMP-9 protein and the seven flavonoids under study, the complex of this protein with 5-(4-phenoxyphenyl)-5-(4-pyrimidin-2-ylpiperazin-1-yl)pyrimidine-2,4,6 (2 h, 3 h)-trione) (compound **8)** was retrieved from PDB under ID 2OVX. Water molecules were removed, and the complexes were prepared in the LEADS-PEP data set using the Protein Preparation Wizard from Maestro Schrödinger. Using the same software, flavonoids were drawn and equilibrated. Hydrogens atoms were added to the protein with PROPKA, and energy minimized with MacroModel with OPLS4 force field.

Molecular docking was carried out using DockThor (https://dockthor.lnCC .br/v2/) (Guedes et al., 2021) employing MMFF94S force field. The box coordinates were defined from compound 8 coordinate center, and the discretization was set to the default value of 0.25 Å. As a control, re-docking was performed by re-coupling the inhibitor in the protein binding site, with the pose achieving an RMSD ≤2.5 Å (Audie and Swanson, 2012). Afterwards, the isolated flavonoids were docked into the MMP-9 binding site, and the results were processed using DTStatistics from DockThormages and analyses of the interactions used Pymol and LigPlot^+^, respectively.

#### 2.7.2 Molecular dynamics simulations

Molecular dynamics simulations were performed with the software GROMACS package. The structures were modeled with Charmm-Gui, using CHARMM36m force field. The protein complexes were solvated in octahedral boxes, by TIP3P solvent model and neutralized with sodium and chloride ions, in the presence of periodic boundary conditions. The minimal margin of the edge distance was 1.2 Å to any solute atom. The overall pressure and temperature were held constant at 1 bar and 300 K, respectively. The systems were equilibrated using a canonical ensemble (NVT) with 5,000 kJ mol-1 positional restrictions applied to the positions of solute molecules. Afterwards, a series of simulations under isobaric-isothermal ensemble (NPT) were performed, decreasing the atomic restrictions, from 5,000 kJ mol^-1^ to 0 kJ mol^-1^ for protein and to 1,000 kJ mol^-1^ for ions and ligands, with 1ns integration time. The Parrinello-Rahman pressure coupling method was utilized in NPT equilibration. Non-bonded interactions were calculated with Particle Mesh Ewald method using a cutoff of 1.0 Å. The trajectory analyses were performed using tools from the GROMACS package, results were plots using Grace software, and representative figures were prepared using Pymol.

### 2.8 *In vitro* cellular assays

#### 2.8.1 Cell culture and treatment

Murine macrophages (RAW264.7 cells) were cultured in DMEM high glucose media, supplemented with 10% fetal bovine serum (FBS), at 37°C in a 5% CO2 incubator (Mensor et al., 2001; Li et al., 2020). Once RAW264.7 cells reached 80% confluency, they were detached and seeded in 96-well plates (1 × 10^5^ cells/well). After 24 h, cells were treated in the presence and absence of LPS at 1 μg/mL with CC extract and tea at 50, 100, or 200 μg/mL, the isolated flavonoids at 50, 100, or 200 μM, dexamethasone at 50 µM (positive control) or the vehicle (negative control), in serum-free media. After 24 h, cell supernatants were collected and stored at −80°C until further use for investigating MMP activity by gelatin zymography.

#### 2.8.2 Cell viability

The Cell Counting Kit-8 (CCK-8) assay was used to quantitatively assess RAW264.7 cells viability after being exposed to the drugs tested following the manufacturer instructions. Briefly, following incubation with various concentrations of the test drugs or the vehicle diluted in serum free culture medium for 24 h, the medium was removed, and cells incubated for another 2 h in the presence of the CCK-8 reagent. Subsequently, a microplate reader (BioTek Instruments, Inc., USA) was employed to measure absorbance at a wavelength of 450 nm.

#### 2.8.3 Gelatinolytic activity

Gelatin zymography was performed to assess MMP-2 and MMP-9 activity after the exposure of the RAW264.7 cells to LPS and the drugs/vehicle as previously described by Medina *et al* (Medina et al., 2006). Cell culture supernatants were subjected to 8% SDS-PAGE polymerized in the presence of gelatin (0.1% wt/v). A sample of conditioned media from HT1080 human fibrosarcoma cells, that contains high amounts of MMP-2 and MMP-9, was loaded as well as internal control. Afterwards, gels were washed three times (20 min each) with Triton X-100 (2.5% v/v) and subsequently incubated for 72 h at 37°C in a buffer containing 2M Tris-HCl (pH 7.5), 0.15M NaCl, 5 mM CaCl_2_, and 0.02% (w/v) sodium azide for allowing the digestion of gelatin by MMP-2 and MMP-9. Following incubation, gels were stained with a Coomassie Brilliant Blue solution for 3 h at room temperature and then distained until transparent bands representing the gelatinolytic activity against the blue background become apparent. Gel images were obtained using the ChemiDoc Documentation System (Bio-Rad, Ireland) and the intensity of the bands quantified using the Biorad image Lab 6.1 software.

#### 2.8.4 Statistical analysis

Statistical analysis and IC_50_ values were carried out using Graph Pad Prism 9.0 software (San Diego, CA, USA). All results are shown as mean ± standard deviation (SD) from at least three independent experiments performed by triplicate. Statistical significance between groups was calculated by one way ANOVA and Dunnett/Duncan’s multiple post-test when appropriated. *p*-values less than 0.05 (*p* < 0.05) were used as the significant level.

## 3 Results

### 3.1 Fingerprinting of the extract by HPLC-ESI-QTOF-MS/MS approach

LC–MS analysis of CC extract demonstrated the presence of highly glycosylated flavonol in addition to fatty acids, glucosinolanes and phenolic acids as previously reported in different caper extracts (Inocencio et al., 2000; Bakr and El Bishbishy, 2016). In total, 24 compounds belonging to different classes were identified by HPLC-ESI-QTOF-MS/MS in CC extract. Data concerning the identification of the peaks are shown in Table 1, in which we report the retention time, electrospray ionization mass spectrometry in negative mode and molecular formula for all of the compounds detected. The structures of all compounds were assessed based on the *m/z* of both precursor ion and fragmentation obtained. Moreover, the spectral data were compared with that reported in the literature, whereas the structures of the isolated compounds were confirmed by NMR.

**TABLE 1:** HPLC-MS determination of major constituents from *CC* extract.

*Rt (min)*	*Ion (+/−)*	*Molecular formula*	*m/z*	*m/z experimental*	*Delta (mmu)*	*DBE*	*MS/MS fragments*	*Proposed compound*	*References*
			*calculated*						
4.3	[M-H]^-^	C_16_H_16_O_5_	287.0925	287.0925	−0.01	9	241.0882, 45.0001	**Racemosin**	Zhou et al. (2011)
4.9	[M-H]^-^	C_10_H_19_NO_9_S_2_	360.0455	360.0440	−3.2	2	259.0135, 195.0329, 96.9603	**Isopropylglucosinolate**	Alsharif et al. (2022)
8.8	[M-H]^-^	C_7_H_6_O_3_	137.0244	137.0243	0.83	5	123.0332, 108.0234	**Protocatechuic aldehyde**	Yang et al. (2010)
8.8	[M-H]^-^	C_11_H_21_NO_9_S_2_	374.0612	374.0628	−5.61	2	259.0121, 195.0346, 96.9605	**2-Butyl glucosinolate**	Alsharif et al. (2022)
9.1	[M-H]^-^	C_9_H_10_O_4_	181.0506	181.0509	−1.47	5	136.9839, 92.9967	**Ethyl 3,4-dihydroxybenzoate**	Yang et al. (2010)
9.4	[M-H]^-^	C_35_H_42_O_9_	605.2756	605.2756	−1.47	15	403.1994, 203.0846	**Scortechinone A**	Reddy et al. (2021)
9.7	[M-H]^-^	C_7_H_6_O_3_	137.0244	137.0245	−0.6	5	93.0368	**Salicylic acid**	Protasiuk and Olejnik (2018)
10.4	[M-H]^-^	C_8_H_8_O_5_	183.0299	183.0307	−4.36	5	168.0089, 124.0181	**Methyl gallate**	Gong et al. (2020)
10.9	[M-H]^-^	C_29_H_23_N_2_O_6_	495.1562	495.1526	7.18	19	-	**Cappariloside**	Wishart et al. (2007)
11.0	[M-H]^-^	C_20_H_32_O_10_	431.1923	431.1940	−4.0	5	385.1899, 179.0573	**Sacranoside A**	Okur et al. (2019)
11.2	[M-H]^-^	C_28_H_32_O_16_	623.1618	623.1669	−8.24	26	315.0516	Isorhamnetin 3*-O-*rutinoside*	Jing et al. (2015)
11.3	[M-H]^-^	C_33_H_40_O_20_	755.2040	755.2071	−4.01	14	300.0298	**Quercetin 3*-O-*** (2G-rhamnosylrutinoside)*	Chen et al. (2015)
11.5	[M-H]^-^	C_27_H_30_O_15_	593.1512	593.1541	−4.89	13	429.0847, 284.0326, 255.0322, 227.0393	Kaempferol 3*-O-*neohesperidoside*	Chen et al. (2015)
11.6	[M-H]^-^	C_33_H_40_O_19_	739.2091	739.2143	−3.91	14	284.0338	**Kaempferol 3*-O-*** (2G-rhamnosylrutinoside)*	Chen et al. (2015)
11.7	[M-H]^-^	C_33_H_40_O_19_	739.2091	739.2115	−3.24	14	284.0329	**Robinin**	Tsiklauri et al. (2011)
11.8	[M-H]^-^	C_27_H_30_O_16_	609.1461	609.1445	2.64	13	300.0265, 271.0248, 225.1131, 178.9964	Quercetin 3*-O-*neohesperidoside*	Chen et al. (2015)
12.3	[M-H]^-^	C_27_H_30_O_16_	609.1461	609.1508	−7.2	13	300.0267, 271.0203, 233,0655	Rutoside*	Abdykerimova et al. (2020)
12.7	[M-H]^-^	C_27_H_30_O_15_	593.1512	593.1525	−2.2	13	284.0353, 255.0314, 227.0344	Kaempferol 3*-O-*rutinoside*	Chen et al. (2015)
20.2	[M-H]^-^	C_18_H_34_O_5_	329.2333	329.2334	−0.16	2	229.1447, 211.1342	**11,12,13-trihydroxy-9-octadecenoic acid**	Lin and Chen (2011)
21.3	[M-H]^-^	C_16_H_32_O_4_	287.2228	287.2231	−1.1	1	269.2106, 241.2148	**3,12-dihydroxypalmitic acid**	Zhang et al. (2016)
22.15	[M-H]^-^	C_12_H_22_O_4_	229.1445	229.1444	0.58	2	211.1329	**Dodecanedioic acid**	Kokotou et al. (2020)
22.8	[M-H]^-^	C_16_H_30_O_4_	285.2071	285.2071	0.12	2	285.2071	**Hexadecanoic acid**	Mok et al. (2016)
26	[M-H]^-^	C_18_H_36_O_4_	315.2541	315.2539	0.58	1	297.2468, 269.2435	**2,3-Dihydroxystearic acid**	Tsami et al. (2022)
27.7	[M-H]^-^	C_18_H_34_O_4_	313.2384	313.238	1.38	2	295.2264, 277.2082	**Octadecanoic acid**	Fuchs et al. (2018)

(DBE, double bond equivalent; Rt, retention time; Delta, difference between experimental and calculated masses; *, indicates the isolated compounds).

**FIGURE 1:**
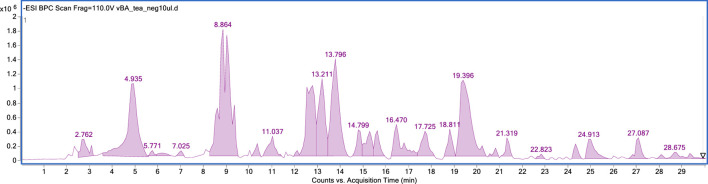
HPLC-MS fingerprint of the *CC* total extract in the negative ionisation mode.

Flavonoids were the major identified components, with a predominance of flavonol class, especially quercetin and kaempferol derivatives. New compounds were identified in this species, such as quercetin and kaempferol triglycosides, as well as their neohesperidoside derivatives. Interestingly, the plant is capable of synthesizing both rutinoside and neohesperidoside derivatives of both flavonols. Additionally, a methylated flavonol glucoside (isorhamnetin-3-rutinoside) was also identified in CC extract, along with two other phenolic compounds: salicylic acid, and methyl gallate. The extract was found to be a rich source of phenolic compounds, making it an ideal choice for subsequent fractionation using counter-current chromatography (see Figure 1).

### 3.2 High speed counter current chromatography (HSCCC) fractionation of CC extract

TLC analysis of CC extract eluted with a mixture of ethyl acetate: formic acid: acetic acid: water (10:2.6:2.6:1) (v/v/v/v), after sprayed with the natural products/PEG reagent (NP/PG), showed the presence of several orange-yellow bands, indicating the presence of flavonoids (chromatogram not shown). Prior to HSCCC separation, a sample of CC extract was subjected to four solvent systems, including hexane, ethyl acetate, methanol, and water (H:E:M:W) (1:6:1:6) (v/v/v/v); hexane, ethyl acetate, butanol, methanol, and water (H:E:B:M:W) (1:6:0.5:1:6) (v/v/v/v); and ethyl acetate, butanol, and water (E:B:W) (4:1:5 and 2:3:5) (v/v/v/v). The upper and lower phases were separated and analysed separately by TLC. The solvent system E: B: W (ethyl acetate: butanol: water) at a ratio of 2:3:5 was selected as it showed the best distribution with phenolic compounds almost equally distributed between the two aqueous lower and organic upper phases.

**FIGURE 2:**
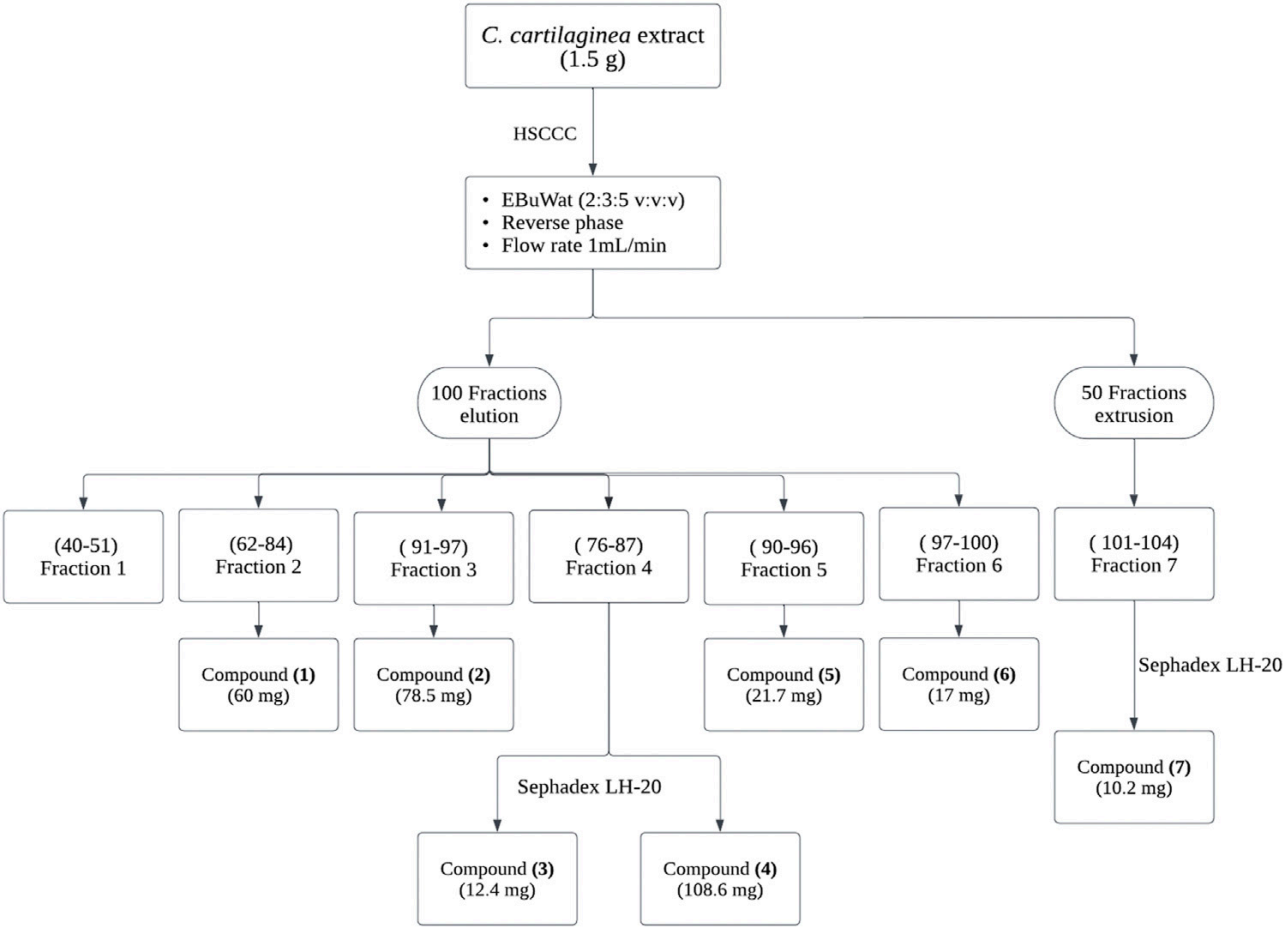
HSCCC Separation procedure of flavonoids from CC extract, followed by their purification using Sephadex LH-20.

HSCCC separation for CC extract was conducted using the selected solvent system in isocratic elution (reversed phase, head to tail). The retention of the stationary phase was 84%–87%. The collected samples were grouped into 7 fractions based on the chromatographic similarities of their constituents (observed Rf values on the TLC plates) (Figure 2). Combined fractions 2, 3, 5, and 6 contained a single flavonoid species, while fractions 4 and 7 required further purification by gel filtration chromatography (Sephadex^®^ LH-20). Spectroscopic analyses allowed the identification of seven main compounds, which were confirmed by comparison with the literature data. The purity of the isolated flavonoids was also checked by HPLC-UV analyses, and all compounds were obtained with a purity over 95*%.*

**FIGURE 3:**
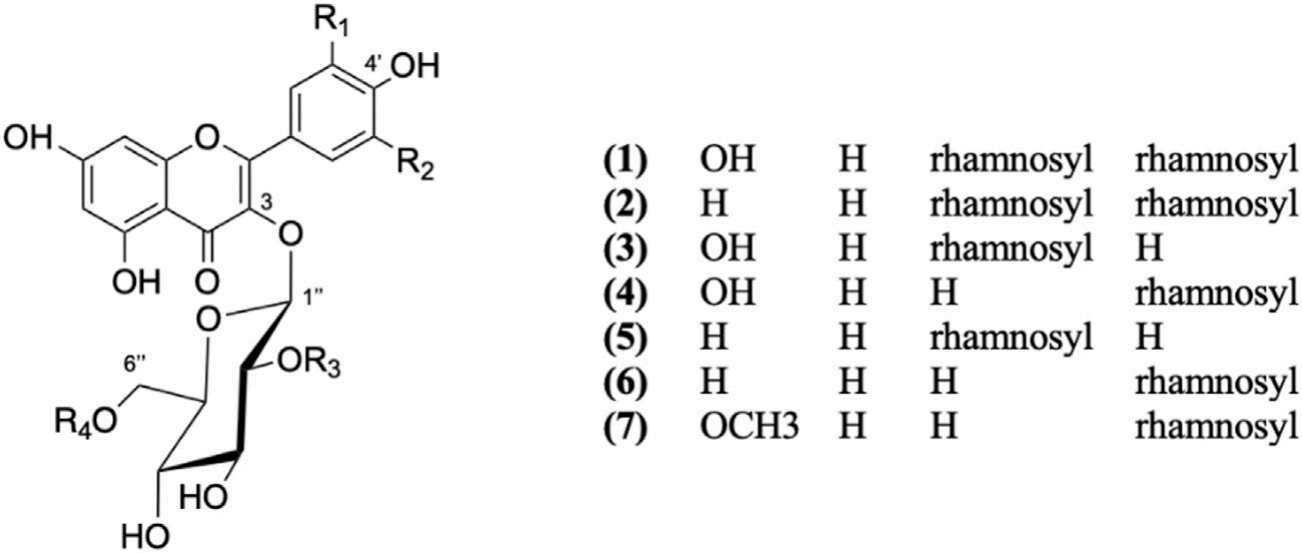
Chemical compounds isolated from CC extract.

Seven phenolic compounds were isolated from CC extract and previously identified by LC-MS (Table 1). The structures of the pure isolates were determined by spectral 1D- and 2D-NMR. Comparison with the literature data allowed to identify the compounds as quercetin 3-(2G-rhamnosylrutinoside) (manghaslin) (Alzweiri et al., 2011), kaempferol 3-(2G-rhamnosylrutinoside) (clitorin) (Chen et al., 2018), quercetin 3- neohesperidoside (Q3-Neo**)** (Köhrmann et al., 2009), rutin (Cui et al., 2017), kaempferol 3- neohesperidoside (K3-Neo) (Galis et al., 1995), nicotiflorin (Nishikaku et al., 2009), isorhamnetin-3-o-rutinoside (narcissin) (Loftus et al., 2000) (Figure 3). The full NMRs and MS/MS fragmentations for each compound are presented as Supplementary Material (Supplementary Table S1; Supplementary Figure S1). The chemical signals obtained for the isolated compounds are described (in ppm) as follows.

**Compound 1:** yellow precipitate: quercetin 3*-O-*(2G-rhamnosylrutinoside (Manghaslin) (60 mg); ESI-MS for C_33_H_40_O_20_ molecular ion, *m/z*; 755 [M-H]-; ^1^H NMR (400 MHz, CD_3_OD) *δ*_H_ 7.62 (1H, d, *J* = 2.2 Hz,H-6′), 7.59 (1H, d, *J* = 2.0 Hz, H-2′), 6.87 (1H, d, *J* = 8.2 Hz, H-5′), 6.37 (1H, d, *J* = 2.2 Hz, H-8), 6.18 (1H, d, *J* = 2.2 Hz, H-6), 5.59 (1H, d, *J* = 7.6 Hz, H-1″), 5.22 (1H, d, *J* = 1.7 Hz, H-1‴), 4.51 (1H, d, *J* = 1.8 Hz, H-1‴'), 4.08 (1H, dd, *J* = 9.6, 6.2 Hz, H-5‴), 4.01 (1H, dd, *J* = 3.4, 1.7 Hz, H-2‴), 3.82 (1H, dd, *J* = 11.5, 1.5 Hz, H-6a''), 3.79 (1H, dd, *J* = 9.4, 3.4 Hz, H-3‴'), 3.65 (1H, dd, *J* = 9.2, 7.6 Hz, H-2″), 3.59 (dd, *J* = 3.5, 1.7 Hz, H-2‴'), 3.54 (1H, t, *J* = 8.9 Hz, H-3″), 3.49 (1H, dd, *J* = 9.5, 3.4 Hz, H-3‴), 3.42 (1H, dq, *J* = 9.7, 6.2 Hz, H-5‴'), 3.36 (1H, t, *J* = 9.8 Hz, H-4″), 3.35–3.30 (m, H-6b''), 3.26 (1H, t, *J* = 9.4 Hz, H-4‴), 3.22 (1H, t, *J* = 9.5 Hz, H-4‴'), 1.08 (3H, d, *J* = 6.2 Hz, H-6‴'), 1.01 (3H, d, *J* = 6.2 Hz, H-6‴).^13^C NMR (100 MHz, CD_3_OD) *δ*_C_ 179.27(C-4), 165.77 (C-7), 163.14 (C-5), 161.22 (C-2), 158.92(C-9), 158.45(C-4′), 134.44(C-6′), 123.53 (C-2′), 123.46 (C-1′), 117.40 (C-3′), 116.05 (C-5′), 105.87 (C-10), 102.65 (C-1‴), 102.25 (C-1‴'), 100.48 (C-1″), 99.77 (C-6), 94.75 (C-8), 80.04 (C-2′), 78.91 (C-3′), 77.08 (C-5′), 74.06 (C-4‴), 73.87 (C-3‴), 72.40 (C-2‴), 72.29 (C-3‴), 72.25 (C-3‴'), 72.14 (C-2‴'), 71.87 (C-4′), 69.96 (C-5‴), 69.72 (C-5‴'), 68.28 (C-6″), 17.82 (C-6‴'), 17.51 (C-6‴). This data is in agreement to that reported by Kazuma et al. (Kazuma et al., 2003).

**Compound 2:** yellow powder: kaempferol 3*-O-*(2G-rhamnosylrutinoside) (Clitorin) (78.5 mg); ESI-MS for C_33_H_40_O_19_ molecular ion, *m/z*; 739 [M-H]-; ^1^H NMR (400 MHz, CD3OD); *δ*_H_ 8.04 (2H, d, *J* = 8.9 Hz, H-6′, 2′), 6.92 (2H, d, *J* = 8.9 Hz, H-5′, 3′), 6.40 (1H, d, *J* = 2.1 Hz, H-8), 6.21 (1H, d, *J* = 2.1 Hz, H-6), 5.62 (1H, d, *J* = 7.5 Hz, H-1″), 5.24 (1H, d, *J* = 1.6 Hz, H-1‴), 4.51 (1H, d, *J* = 1.7 Hz, H-1‴'), 4.07 (1H, dq, *J* = 9.7, 6.1, H-5‴), 4.02 (1H, dd, *J* = 1.6, 3.4, H-2‴), 3.81 (1H, dd, *J* = 12.5, 1.0, H-6a''), 3.79 (1H, dd, *J* = 9.7, 3.4, H-3‴), 3.59 (1H, dd, *J* = 8.9, 7.6, H-2″), 3.54 (1-H, t, *J* = 8.9, H-3″), 3.23 (1H, t, J = 8.9, H-4″), 3.34 (m-H-5″), 3.37 (1H, dd, J = 6.1, 12.5, H-6b''), 3.33 (1H, t, J = 9.7, H-4‴), 3.41 (1H, dq, *J* = 6.2, 9.5 Hz, H-5‴'), 3.28 (1H, t, *J* = 9.5 Hz, H-4‴'), 3.57 (1H, dd, *J* = 1.5, 3.4 Hz, H-2‴'), 3.47 (1H, dd, *J* = 3.4, 9.5 Hz, H-3‴'), 1.10 (3H, d, *J* = 6.2 Hz, H-6‴'), 1.00 (3H, d, *J* = 6.2 Hz, H-6‴). ^13^C NMR (100 MHz, CD3OD) *δ*_C_ 179.28(C-4), 165.61(C-7), 163.14(C-5), 161.22(C-2), 159.03(C-9), 158.47(C-4′), 134.32(C-6′), 132.15(C-2′), 123.14(C-1′), 116.15(C-3′), 105.93(C-10), 102.59(C-1‴), 102.27(C-1‴'), 100.45(C-1″), 99.77(C-6), 94.75(C-8), 79.87(C-2′), 78.91(C-3′), 77.07(C-5′), 74.02(C-4‴), 73.80(C-3‴), 72.72(C-2‴), 72.39(C-3‴), 72.29(C-3‴'), 72.09(C-2‴'), 71.92(C-4′), 69.90(C-5‴), 69.73(C-5‴'), 68.28(C-6″), 17.83(C-6‴'), 17.54(C-6‴). This data is in agreement to that reported by Kazuma et al. (Kazuma et al., 2003).

**Compound 3:** yellow powder: quercetin 3*-O-*neohesperidoside (Q3-Neo) (12.4 mg); ESI-MS for C_27_ H_30_O_16_ molecular ion, *m/z*; 593 [M-H]-; ^1^H NMR (400 MHz, CD3OD) *δ*_H_ 7.61 (1H, d, *J* = 2.2 Hz, H-2′), 7.60 (1H, dd, *J* = 8.3, 2.0 Hz, H-6′), 6.87 (1H, d, *J* = 8.5 Hz, H-5′), 6.36 (1H, d, *J* = 2.1 Hz, H-8), 6.17 (1H, d, *J* = 2.1 Hz, H-6), 5.73 (1H, d, *J* = 7.6 Hz, H-1″), 5.22 (1H, d, *J* = 1.7 Hz, H-1‴), 4.03 (1H, dq, *J* = 9.6, 5.9, H-5‴), (1H, dd, *J* = 3.4, 1.5 Hz, H-2‴), 3.77 (1,H, dd, *J* = 9.4, 3.4 Hz, H-3‴), 3.73 (1H, dd, *J* = 12.1, 2.3 Hz, H-6a''), 3.63 (1H, dd, *J* = 7.6, 9.4 Hz, H-2″), 3.55 (1H, t, *J* = 9.4 Hz, H-3″), 3.54 (1H, dd, *J* = 12.1, 5.6 Hz, H-6b''), 3.46 (dd, *J* = 8.0, 7.4 Hz, 1H), 3.34 (1H, t, *J* = 9.4 Hz, H-4‴), 3.33 (1H, t, *J* = 9.4 Hz, H-4″), 0.99 (3H, d, *J* = 6.2 Hz, H-1‴). ^13^C NMR (100 MHz, CD_3_OD) *δ*_C_ 179.35(C-4), 165.60(C-7), 163.18(C-5), 158.44(C-2), 158.38(C-9), 149.54(C-4′), 146.00(C-3′), 134.70(C-3), 123.46(C-6′), 123.22(C-1′), 117.21(C-2′), 115.98(C-5′), 105.45(C-10), 102.94(C-1‴), 100.35(C-1″), 99.66(C-6), 94.20 (C-8), 80.13 (C-2″), 78.94 (C-3″), 78.32 (C-5″), 74.06(C-4‴), 72.41(C-2‴), 72.31(C-3‴), 71.72(C-4″), 69.96(C-5‴), 62.57(C-6″),17.46 (C-6‴). This data is in agreement to that reported by Kazuma et al. (Kazuma et al., 2003).

**Compound 4:** yellow crystals: quercetin 3*-O-*rutinoside (rutin) (108.6 mg); ESI-MS for C_27_H_30_O_16_ molecular ion, *m/z*; 609 [M-H]-; ^1^H NMR (400 MHz, CD3OD)); *δ*_H_ 7.67 (1H, d, *J* = 2.2 Hz, H-2′), 7.63 (1H, dd, *J* = 8.3, 2.0 Hz, H-6′), 6.87 (1H, d, *J* = 8.5 Hz, H-5′), 6.39 (1H, d, *J* = 2.1 Hz, H-8), 6.19 (1H, d, *J* = 2.1 Hz, H-6), 5.09 (1H, d, *J* = 7.6 Hz, H-1″), 4.52 (1H, d, *J* = 1.7 Hz, H-1‴), 3.81 (1H, dd, *J* = 11.1, 1.4 Hz, H-6a''), 3.63 (1H, dd, *J* = 3.4, 1.7 Hz, H-2‴), 3.54 (1H, dd, *J* = 9.5, 3.4 Hz, H-3‴), 3.46 (1H, dd, *J* = 8.0, 7.4 Hz, H-5″), 3.44 (1H, dq, *J* = 9.6, 6.9, H-5‴), 3.40 (1H, t, *J* = 9.4 Hz, H-3″), 3.38 (1H, dd, *J* = 11.1, 1.4 Hz, H-6b''), 3.32 (1H, ddd, *J* = 6.1, 1.0 Hz, H-5″), 3.26 (1H, t, *J* = 9.4 Hz, H-4″), 3.27 (1H, t, *J* = 9.6 Hz, H-4‴), 1.13 (3H, d, *J* = 6.2 Hz, H-6‴). ^13^C NMR (100 MHz, CD_3_OD) *δ*_C_ 179.31(C-4), 167.09(C-7), 162.96(C-5), 159.21 (C-9), 158.61(C-2), 149.88(C-4′), 145.88(C-3), 135.17(C-3), 123.54(C-6′), 123.12(C-1′), 117.21(C-2′), 116.06(C-5′), 105.34(C-10), 102.44(C-1‴), 95.09(C-8), 78.22(C-3″), 77.24(C-5″), 75.73(C-2″), 73.94(C-4‴), 72.24(C-3‴), 72.11(C-2‴), 71.40(C-4″), 69.71(C-5‴), 68.56(C-6″), 17.89(C-6‴). This data is in agreement to that reported by Kazuma et al. (Kazuma et al., 2003).

**Compound 5:** yellow powder: kaempferol 3*-O-*neohesperidoside (K3-Neo) (21.7 mg); ESI-MS for C_27_H_30_O_15_ molecular ion, *m/z*; 593 [M-H]-; ^1^H NMR (400 MHz, CD3OD) *δ*_H_ 8.07 (2H, d, *J* = 8.9 Hz, H-6′, 2′), 6.89 (2H, d, *J* = 8.9 Hz, H-5′, 3′), 6.39 (1H, d, *J* = 2.1 Hz, H-8), 6.20 (1H, d, *J* = 2.1 Hz, H-6), 5.12 (1H, d, *J* = 7.3 Hz, H-1″), 4.52 (1H, d, *J* = 1.7 Hz, H-1‴), 3.81 (1H, dd, *J* = 10.9, 1.4 Hz, H-6a), 3.63 (1H, dd, *J* = 3.4, 1.6 Hz, H-2‴), 3.52 (1H, dd, *J* = 9.5, 3.4 Hz, H-3‴), 3.44 (1H, dq, *J* = 9.6, 6.2, H-5‴), 3.42 (1H, d, *J* = 7.3, 5.5Hz, H-2″), 3.40 (1H, t, *J* = 8.8 Hz, H-3″), 3.32 (1H, ddd, *J* = 1.0, 6.1, 8.0 Hz, H-5″), 3.24 (1H, t, *J* = 8.8 Hz, H-4″), 3.39–3.34 m), 3.33 (d, *J* = 1.6 Hz, 1H), 3.27 (1H, t, *J* = 2.9 Hz, H-4‴), 3.25 (d, *J* = 2.8 Hz, 1H), 1.12 (3H, d, *J* = 6.2 Hz, CH_3_-1‴). ^13^C NMR (100 MHz, CD_3_OD) *δ*_C_ 179.33(C-4), 165.06(C-7), 162.99(C-5), 161.55 (C-2), 159.30(C-4′), 158.65(C-9), 135.49(C-3), 132.37(C-2′), 132.36(6′), 122.78(C-1′), 116.15(C-3′), 116.15(C-5′), 105.41(C-10), 104.69 (C-1″), 102.44(C-1‴), 100.27(C-6), 95.12(C-8), 78.17(C-5″), 77.24(C-5″), 75.77(C-2″), 73.90(C-4‴), 72.29(C-3‴), 72.10(C-2‴), 71.45(C-4″), 69.74(C-5‴), 68.57(C-6″), 17.92(C-6‴). This data is in agreement to that reported by Kazuma et al. (Kazuma et al., 2003).

**Compound 6:** yellow powder: kaempferol 3*-O-*rutinoside (nicotiflorin) (17 mg); ESI-MS for C_27_ H_30_O_15_ molecular ion, *m/z*; 593 [M-H]-; ^1^H NMR (400 MHz, CD_3_OD)) *δ*_H_ 8.07 (2H, d, *J* = 8.9 Hz, H-6′, 2′), 6.89 (2H, d, *J* = 8.9 Hz, H-5′, 3′), 6.39 (1H, d, *J* = 2.1 Hz, H-8), 6.20 (1H, d, *J* = 2.1 Hz, H-6), 5.12 (1H, d, *J* = 7.3 Hz, H-1″), 4.52 (1H, d, *J* = 1.7 Hz, H-1‴), 3.81 (1H, dd, *J* = 10.9, 1.4 Hz, H-6a), 3.63 (1H, dd, *J* = 3.4, 1.6 Hz, H-2‴), 3.52 (1H, dd, *J* = 9.5, 3.4 Hz, H-3‴), 3,44 (1H, dq, *J* = 9.6, 6.2, H-5‴), 3.42 (1H, d, *J* = 7.3, 5.5Hz, H-2″), 3.40 (1H, t, *J* = 8.8 Hz, H-3″), 3.32 (1H, ddd, *J* = 1.0, 6.1, 8.0 Hz, H-5″), 3.24 (1H, t, *J* = 8.8 Hz, H-4″), 3.39–3.34 m), 3.33 (d, *J* = 1.6 Hz, 1H), 3.27 (1H, t, *J* = 2.9 Hz, H-4‴), 3.25 (d, *J* = 2.8 Hz, 1H), 1.12 (3H, d, *J* = 6.2 Hz, CH_3_-1‴). ^13^C NMR (100 MHz, CD_3_OD) *δ*_C_ 179.33(C-4), 165.06(C-7), 162.99(C-5), 161.55 (C-2), 159.30(C-4′), 158.65(C-9), 135.49(C-3), 132.37(C-2′), 132.36(6′), 122.78(C-1′), 116.15(C-3′), 116.15(C-5′), 105.41(C-10), 104.69 (C-1″), 102.44(C-1‴), 100.27(C-6), 95.12(C-8), 78.17(C-5″), 77.24(C-5″), 75.77(C-2″), 73.90(C-4‴), 72.29(C-3‴), 72.10(C-2‴), 71.45(C-4″), 69.74(C-5‴), 68.57(C-6″), 17.92(C-6‴). The NMR data is in agreement to that reported by (Cedeño et al., 2019).

**Compound 7:** Bright yellow amorphous powder: Isorhamnetin 3*-O-*rutinoside (Narcissin) (10.2 mg); ESI-MS for C_28_H_32_O_16_ molecular ion, *m/z*; 623 [M-H]-; ^1^H NMR (400 MHz, CD_3_OD) *δ*_H_ 7.97 (d, *J* = 2.1 Hz, H-2′), 7.65 (1H, dd, *J* = 8.5, 2.1 Hz,H-6′), 6.44 (1H, d, *J* = 2.1 Hz, H-8), 6.23 (1H, d, *J* = 8.5 Hz, H-6), 5.26 (1H, d, *J* = 7.2 Hz, H-1″), 4.55 (1H, d, *J* = 1.8 Hz, H-1‴), 3.97 (3H, s, OMe-3′), 3.82 (1H, dd, *J* = 11.2, 1.5 Hz, H-6″), 3.63 (1H, dd, *J* = 3.4, 1.6 Hz, H-2‴), 3.51 (1H, dd, *J* = 3.4, 1.6 Hz, H-3‴), 3.44 (1H, dd, *J* = 9.4, 6.4 Hz, H-5‴), 3.42 (1H, dd, *J* = 7.3, 5.5 Hz, H-2″), 3.40–3.33 (m, H-3″,5″), 3.27 (1H, t, *J* = 9.5 Hz, H-4‴, 3.25 (1H, d, *J* = 2.8 Hz, H-4″), 1.12(3H, d, *J* = 6.2 Hz, 6‴). ^13^C NMR (100 MHz, CD_3_OD) *δ*_C_ 179.30 (C-3), 166.25(C-7), 162.99 (C-5), 161.55, 150.83(C-4’), 158.83(C-2), 158.51(C-9), 148.30 (C-3′), 135.49(C-3), 123.95(C-6′), 123.03 (C-1′), 116.15(C-5′), 114.51(C-2′), 105.41(C-10), 104.69(C-1″), 102.44(C-1‴), 99.27(C-6), 94.91(C-8), 78.14 (C-5″), 75.87(C-3″), 73.83(C-4‴), 72.23(C-3‴), 72.09(C-2‴), 71.58(C-4″), 69.74, 68.48(C-6″), 56.41(OMe-3′), 17.30 (C-6‴) (Ganbaatar et al., 2015).

### 3.3 Antioxidant activity assays

**FIGURE 4:**
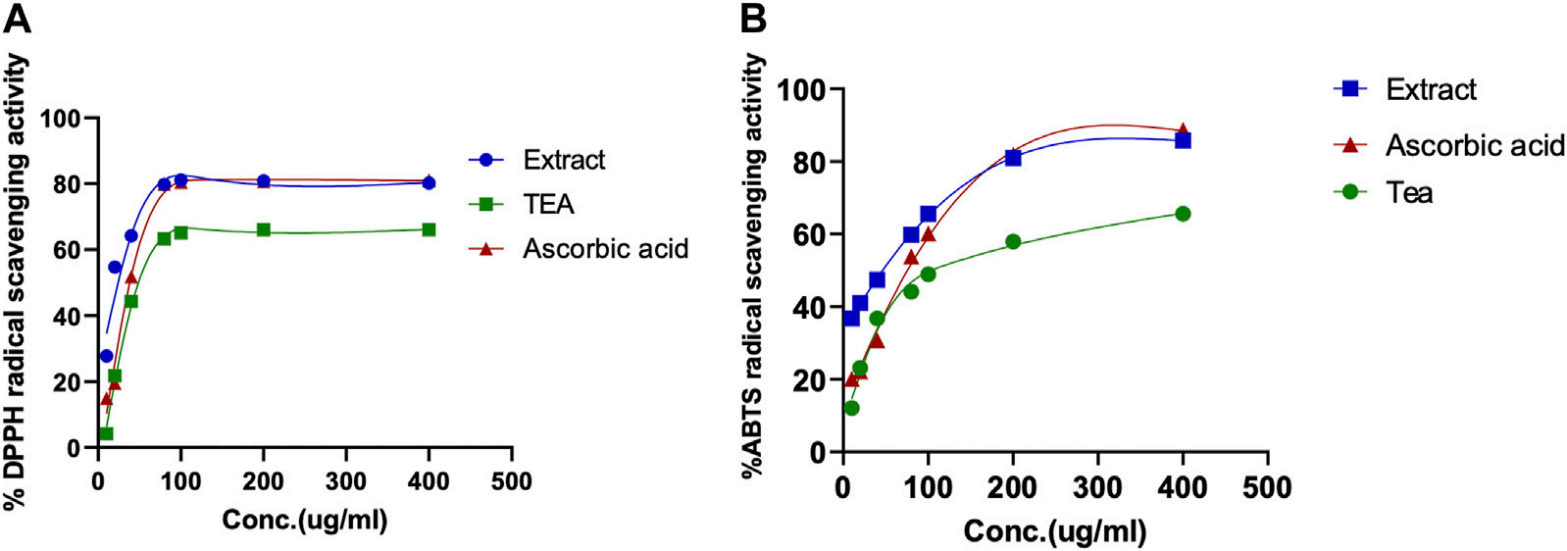
Determination of the antioxidant activity of CC extract and tea. **(A)** 1,1-Diphenyl-2-picrylhydrazyl (DPPH), radical scavenging assay; **(B)** 2,2-azinobis (3-ethylbenzothiazoline-6-sulfonate) (ABTS) radical scavenging assay. Ascorbic acid was used as positive control.

In order to determine the potential antioxidant activity of the CC tea and extract, their free radical scavenging activity against stable DPPH and ABTS was measured using a spectrophotometer. The data obtained is represented and summarized in Figure 4 and Table 2.

**TABLE 2:** Total phenolics and flavonoid content and free radical scavenging activity of CC extract and tea.

Sample	Total phenolics ^(*)^	Total flavonoids ^(**)^	IC_50_ values (µg/mL)	
			DPPH scavenging	ABTS scavenging
CC tea	514.6 ± 0.42	70.8 ± 1.22	48.5 ± 4.44	103.8 ± 1.18
CC extract	734.6 ± 0.19	195.3 ± 0.39	23.7 ± 1.42	46.5 ± 2.61
Ascorbic acid	-	-	38.08 ± 0.13	75.76 ± 0.11

(*) µg GAE/mg of dry weight (**) µg QEs/mg of dry weight.

The data are given as mean ± standard deviation (SD) of triplicate experiments.

### 3.4 Prospection of potential biological targets

**FIGURE 5:**
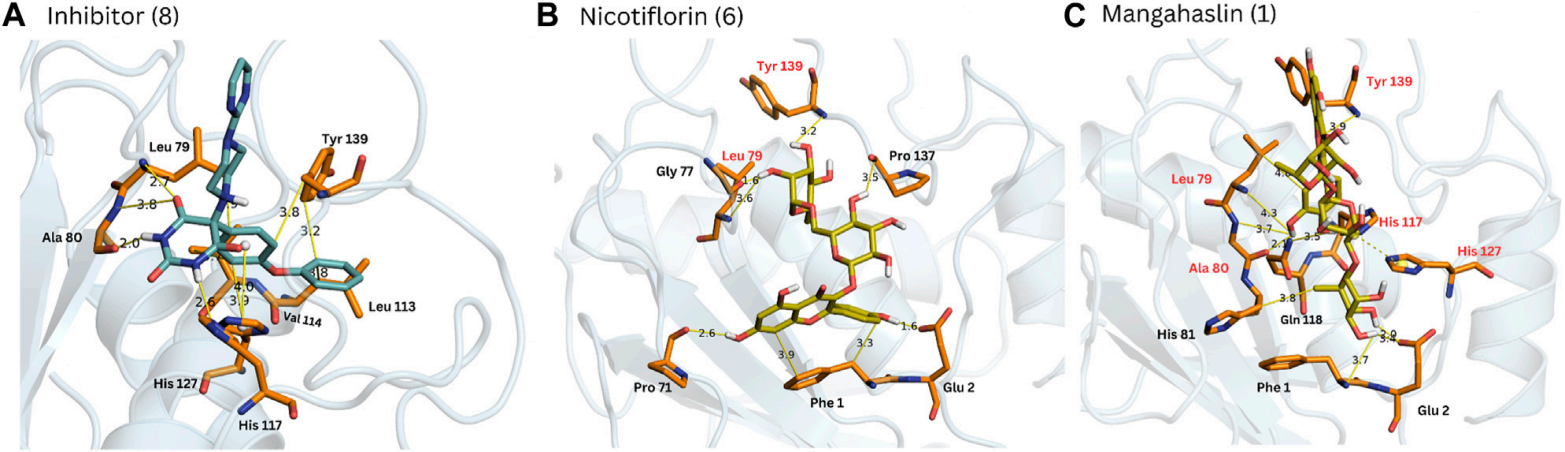
Complexes obtained for compounds 8, 6 and 1 from docking calculations. The main interactions with MMP-9 amino acid residues are indicated. Red residues in **(B, C)** are common to the reference compound 8 **(A)**.

A combination of docking and molecular dynamics were employed, probing the binding and stability of the isolated flavonoids to the MMP-9 active site. Accordingly, most compounds showed a binding energy similar to compound 8 (Table 3), as well as similar interactions with key amino acid residues, as it was possible to observe several chemical interactions between the ligands and amino acid residues Tyr139 and Leu79 (Figure 5). Additionally, the docking obtained complexes were further refined through molecular dynamics simulations, from which the compounds nicotiflorin, manghaslin, clitorin, rutin and Q3-Neo presented the most stable binding (Table 3). These combined results suggested that the studied flavonoids, mainly the five mentioned above, have a potential to inhibit MMP-9. See Supplementary Figures S2–S6.

**TABLE 3:** Modelling of the compounds binding to MMP-9 through a combination of docking and molecular dynamics calculations. The compounds are ranked by the DockThor docking score. Additionally, the average distance to the center of mass of the protein and ligand during the MD simulations are presented, as well as the protein RMSD indicating, respectively, the stability on the ligand binding and on the protein structure.

Molecule	Score (kcal.mol^-1^)	Distance (nm)	RMSD (nm)
Lim et al., (2019) Inhibitor	−10	0.19 ± 0.02	0.26 ± 0.04
Nishikaku et al., (2009) Nicotiflorin	−9.8	0.18 ± 0.01	0.15 ± 0.04
Alzweiri et al., (2011) Manghaslin	−9.7	0.18 ± 0.01	0.21 ± 0.03
Chen et al., (2018) Clitorin	−9.7	0.18 ± 0.02	0.26 ± 0.03
Cui et al., (2017) Rutin	−9.5	0.19 ± 0.01	0.24 ± 0.03
Köhrmann et al., (2009) Q3-Neo	−9.3	0.18 ± 0.01	0.21 ± 0.04
Loftus et al., (2000) Narcissin	−8.7	0.49 ± 0.41	0.27 ± 0.04
Galis et al., (1995) K3-Neo	−8.6	0.26 ± 0.19	0.21 ± 0.04

#### 3.4.1 Evaluation of the cytotoxic effect of CC in RAW264.7 cells

**FIGURE 6:**
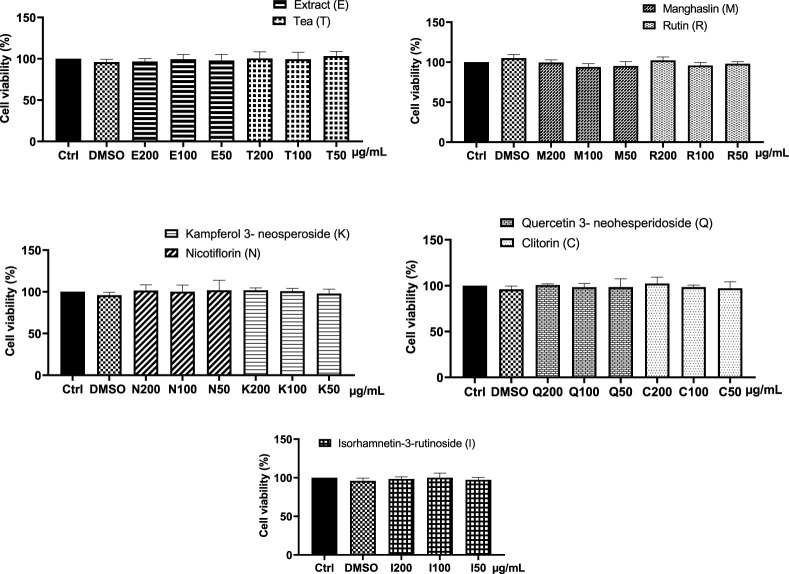
Effect of CC tea, extract, and the isolated compounds on cell viability in RAW 264.7 macrophages. Data represent the mean ± SD of at least three independent experiments. One way ANOVA (*p* = 0.8575).

With the aim of assessing the potential cytotoxic effect of CC extract, tea, and the isolated flavonoids on RAW 264.7 macrophages, cell viability following 24 h of incubation with various concentrations of the compounds was determined using the CCK-8 assay. The results obtained showed that CC tea and extract from 50–200 μg/mL and the isolated flavonoids from 50–200 μM had no cytotoxic effect on RAW264.7 macrophages with cell viabilities over 90% for all the concentrations tested (Figure 6).

### 3.5 Gelatinolytic activity

Following the *in silico* assays that suggested that the flavonoids could potentially modulate MMP-9, the effect of the CC extract, tea and the isolated flavonoids on MMP-9 activity from murine macrophages (RAW 264.7 cells) in the presence of LPS was studied by gelatin zymography.

**FIGURE 7:**
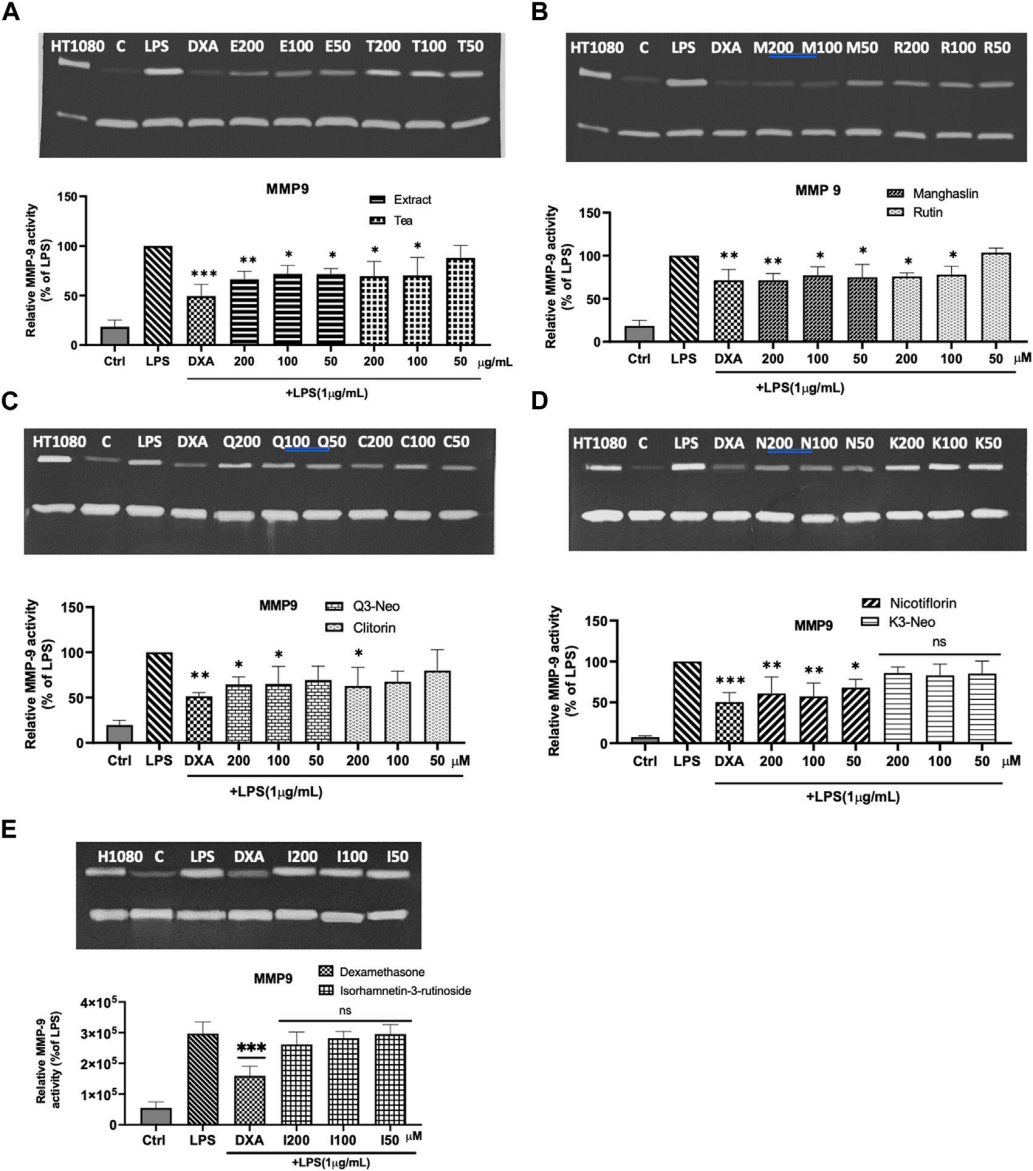
Modulation of MMP-9 activity in LPS stimulated macrophages. **(A)** Representative zymogram and statistical analysis on the effect of tea (T) and extract (E) on MMP 9. **(B)** Representative zymogram and statistical analysis on the effect of manghaslin (M) and rutin (R) on MMP9. **(C)** Representative zymogram and statistical analysis on the effect of Q3-Neo (Q) and clitorin (C) on MMP9. **(D)** Representative zymogram and statistical analysis on the effect of nicotiflorin (N) and K3-Neo (K) on MMP9. **(E)** Representative zymogram and statistical analysis on the effect of isorhamnetin-3-O-rutinoside (I) on MMP9. The relative activity of MMP-9 activity was quantified by densitometry and normalized to the values obtained in LPS treated cells. Each bar represents the mean ± SE of three independent experiments. One way ANOVA and Dunnett post-test; *p < 0.1 **p < 0.01, ***p < 0.001 vs LPS control.

As depicted in Figure 7, RAW 264.7 cells that were not exposed to LPS displayed a very weak gelatinolytic activity at 92 kDa, (corresponding to MMP-9 activity). However, after 24 h of incubation with LPS (1 μg/mL), the conditioned media from the cells exhibited a significant increase in MMP-9 activity. As shown in Figure 7A, the impact of LPS on MMP-9 activity was significantly counteracted by both the extract and the tea. All the purified compounds, except K3-Neo (Figure 7D) and isorhamnetin-3-rutinoside (Figure 7E) were able to downregulate MMP-9 activity (Figures 7B–D). Among the tested flavonoids, manghaslin and nicotiflorin displayed a significant decrease of MMP-9 activity in a dose-dependent manner and their effect was comparable to the one exerted by dexamethasone at 50 μM (Figures 7B, D). Rutin and clitorin also modulated MMP-9 activity but only at high concentrations (Figures 7B, C). The activity of MMP-2, which is constitutively expressed, was unaffected by LPS and its activity remained consistent when cells were co-incubated with the compounds tested (Supplementary Figure S7). This finding is consistent with the results reported by Woo et al. and Chae et al. (Woo et al., 2004; Chae et al., 2012).

## 4 Discussion

The pharmacological activities of the *Capparis* species are mainly attributed to its polyphenolic contents. CC is a member of *Capparis* species which has been traditionally used as tea to treat inflammatory conditions. Although extensively used in traditional medicine, a gap in the research of the chemical compounds present in the tea of this plant still remains. A very limited number of studies on the phenolic composition of this species (Galib and Algfri, 2016) has been published. In this work, CC extract was examined using HPLC-ESI-QTOF-MS/MS, revealing a rich presence of phenolic compounds, with flavonoids being the primary constituents. HPLC-ESI-QTOF-MS/MS was chosen initially because it represented a quick and easy way to profile the extract. Consequently, the extract underwent further fractionation by HSCCC, and the isolated compounds were assessed for their antioxidant and anti-inflammatory activities, along with the tea, where they were also present.

HSCCC was successfully applied to preparative isolation of the main phenolics from CC. HSCCC is different from other traditional chromatographic because its stationary phase and mobile phase are both liquids. As a separation technique, HSCCC provided significant advantages over other traditional separation methods, including non-contamination, higher separation efficiency, low sample loss, high recovery, and high separation volume. A critical step for successful HSCCC separation is the selection of a suitable solvent system. The two-phase solvent system must ensure the absence of sample decomposition or denaturation, offer sufficient sample solubility, exhibit appropriate partition coefficient K) values for the target compound (typically falling within the range of 0.2–5), and maintain the stationary phase’s stability (with the solvent system settling within ≤30 s) (Oka et al., 1991). From the HPLC-ESI-QTOF-MS/MS analysis it was found out the CC extract predominantly contains quercetin and kaempferol glycosides which belong to a class of flavonoids known as flavonol glycoside. This class of compounds are known to be highly polar compounds and freely dissolve in water. Several typical biphasic solvent systems for separation of component with high polarity are available, such as hexane-ethyl acetate-methanol-water, hexane-ethyl acetate-butanol-methanol-water, ethyl acetate–n-butanol–water. We investigated the suitability of those solvent system by TLC. The results showed that a large number of flavonoids were partitioned into the aqueous phase for most of the above solvent systems because of its high polarity. The best result was achieved with a mixture of ethyl acetate-butanol-water (2:3:5, v/v/v), with phenolic compounds almost equally distributed between the two aqueous lower and organic upper phases. This solvent system was successfully used before to isolate flavonoid glycosides from *Lotus plumule* (Wu et al., 2018)*.* Thus, the solvent system ethyl acetate–n-butanol–water (2:3:5, v/v/v) was selected.

Under the conditions used, seven flavonoids were isolated from the CC extract. Spectroscopic analyses allowed the identification of the compounds as, manghaslin, rutin, clitorin, Q3-Neo, K3-Neo, nicotiflorin and narcissin (Figure 3). These compounds have been isolated from this species for the first time, except for nicotiflorin, and rutin which had previously been isolated from CC ethanolic extract from Egypt, alongside quercetin-7-O-rutinoside and quercetin-3-glucoside-7-O-rhamnoside (Sharaf et al., 1997). Rutin has been reported as the most abundant flavonoid in caper leave, fruits and buds (fresh and pickled) (Rodrigo et al., 1992; Nabavi et al., 2016). Nicotiflorin has also been found in the leaves extract of *C. spinosa* and *C. decidua* (Sharaf et al., 1997). Methoxylated flavonoids such as narcissin, wogonin, oroxylin A, have been reported in *C. spinos*a fruit and the whole plant of *C. himalayensis* (Li et al., 2008).

The antioxidant properties of CC tea and extract, were tested in three ways: their total phenolic, flavonoid content, and their antioxidant capacity. It is very important to mention that the antioxidant activity tested herein bears no translational biological value *per se*; it was done so more for quality assurance and comparison with previously published data. The extracts and tea of CC showed a high antioxidant profile as evidenced by the determination of total phenolics and flavonoid equivalent and their radical scavenging activity. The phenolic content of the tested samples (CC tea and extract) was expressed as gallic acid equivalents and it was found to be higher in the extract (734.66 ± 0.01 μg GAE/mg) than in the tea (514.67 ± 0.011 μg GAE/mg). The extract also yielded the highest amount of flavonoids with 195.31 ± 0.002 µg QEs/mg (Table 2). Upon comparing the total polyphenol and flavonoids content of our leaves extract with that of the extract derived from CC leaves in Jordan, which was reported to be 233.50 ± 1.18 μg (GAE/mg) by Al-Qudah et al., we determined that our extract exhibits a higher phenolic content (Al-Qudah et al., 2018). On the other hand, the total flavonoid content was higher in CC extract from Jordan, where the content was reported as 966.61 ± 3.40 μg quercetin equivalent/mg of dried leaf extract (Al-Qudah et al., 2018). These variations and differences in total phenol and flavonoid contents might be due to geographical factors and the use of distinct processing methods. The antioxidant activity of CC extracts and tea were evaluated by two different methods: DPPH and ABTS free radical scavenging activities (Figures 4A, B). In agreement with the phenolics content, CC leaves extract showed a strong antioxidant activity by inhibiting free radicals when compared with the standard L-ascorbic acid, whereas the tea showed a moderate activity in this regard (Table 2). However, the extract of CC from Jordan by Al-Qudah’s research team demonstrated lower capacity compared to our extract (IC_50_: DPPH: 150 ± 0.001; ABTS: 90 ± 0.01 μg/mL) (Al-Qudah et al., 2018).

To evaluate the affinity and potential modulation of MMP-9 activity of the CC extract and the isolated flavonoids, a hybrid *in silico* and *in vitro* approach was used. The *in silico* analysis shown that all flavonoids had a significant predicted affinity to the protein, near to control (−10 kcal.mol^-1^), and many amino acids that were present in the MMP-9-inhibitor interaction were also present in the molecular docking with the flavonoids, suggesting that there is a similarity of biological function between both. This result is in agreement with the molecular dynamics data, where manghaslin, clitorin, Q3-Neo, rutin and nicotiflorin, produced the most negative score, which suggests a greater similarity in interaction behavior with the inhibitor, and also presented a trajectory that suggests greater stability of interaction with the protein, demonstrated by the uninterrupted association between the flavonoid and the MMP-9. The inhibitory effect of some flavonoids on MMPs, mainly 1, 2, 3 and 9, has already been reported in the literature (Hilliard et al., 2020; Kubina et al., 2021; Zhang et al., 2022), that showed the potential inhibitory effect of kaempferol and quercetin in the BRENDA enzymes database (Chung et al., 2009; Zhang et al., 2022).

We have demonstrated that the treatment of LPS stimulated macrophages with CC extract, tea and its flavonoids significantly reduced their MMP-9 activity as demonstrated by gelatin zymography. This is not surprising as flavonoids have been previously recognized for their ability to downregulate the synthesis of MMPs in numerous cell types, such as macrophages, fibroblasts (Lin et al., 2003) or vascular smooth muscle cells (Kim, 2003; Moon et al., 2003) among others and are known to modulate the levels of MMPs through various mechanisms. For example, quercetin inhibits the invasion of murine melanoma cells by reducing pro-MMP-9 through the PKC pathway (Zhang et al., 2004). Furthermore, given that specific MMPs can be activated by oxidative stress (Fu et al., 2001), the widely acknowledged antioxidative properties of flavonoids (Rice-Evans, 2004) might also contribute to effectively restraining such activation mechanisms. Another potential mechanism, is related to the direct inhibition of the enzymatic activity by flavonoids that has been reported for certain enzymes like leucine aminopeptidase, aminopeptidase M and carboxypeptidase A, but also for MMP-2 and -9 (Parellada et al., 1998; Ende and Gebhardt, 2004).

MMP-9 is known to play an important role in rheumatoid arthritis and osteoarthritis, contributing to synovial fibroblast survival, proliferation, migration and invasion (Xue et al., 2014). In a recent publication by Li *et al.* the authors have reviewed the data available in the literature where ‘herbal medicines were found to inhibit the inflammatory process associated to rheumatoid arthritis by regulating MMPs and protecting join structures’ (Li et al., 2022). There is no doubt that the inhibition of MMP-9 can be beneficial for the management of inflammatory conditions and can justify the traditional use of CC tea in the treatment of rheumatism, arthritis, and gout, which are usually managed by, among others, anti-inflammatory drugs. However, under some circumstances, off-target effects or the need for high doses to control the disease may lead to undesirable side effects. A suitable approach that could overcome those problems is using nanocarriers that, targeting inflammation, allow the release of the drug locally, reducing side effects. There are multiple investigations where not only ‘classical synthetic drugs’ but “natural products” have been formulated using nanotechnology to treat different conditions such as cancer, cardiovascular diseases, or inflammation (Hesari et al., 2021; An et al., 2023; Hussain et al., 2023; Tomou et al., 2023; Zandieh et al., 2023).

In this study, we have shown that CC extract and tea are rich in phenolic compounds and that they possess a strong antioxidant activity. The *in silico* model, presented excellent predictions of the relative inhibitory effect of the purified flavonoids on MMP-9, as their biological activity was consistent with the compounds’ binding affinity. Further studies are warranted to investigate further the mechanisms involved in MMP-9 modulation by CC flavonoids and their potential application for the treatment of inflammatory diseases including their formulation as nanodrug delivery systems.

## 5 Conclusion

In this study, seven flavonoid glycosides from the extract of CC leaves, manghaslin, clitorin, rutin, kaempferol 3- neohesperidoside, Quercetin 3- neohesperidoside, nicotiflorin, and narcissin were separated for the first time by one-step HSCCC separation. The high phenolic content of the CC extract, their antioxidant effect and the modulation of the MMP-9 activity demonstrated in this work may explain the potential anti-inflammatory effect of CC and justify the ethnopharmacological use of these species in inflammatory articular diseases.

## Data Availability

The original contributions presented in the study are included in the article/Supplementary Material, further inquiries can be directed to the corresponding author.
