# Supplementary material for: Capparis cartilaginea decne (capparaceae): isolation of flavonoids by high-speed countercurrent chromatography and their anti-inflammatory evaluation
Source: Front Pharmacol. 2023 Oct 19;14:1285243. doi: 10.3389/fphar.2023.1285243 (PMC10620733; doi:10.3389/fphar.2023.1285243)

Supplementary Material

Table 1. The MS/MS fragmentation spectra of the components that were identified in the studied samples by HPLC-MS

<table>
<tr><td colspan="2">The MS/MS spectra of the tentatively identified compounds</td></tr>
<tr><td>11,12,13-trihydroxy-9-octadecenoic acid</td><td>
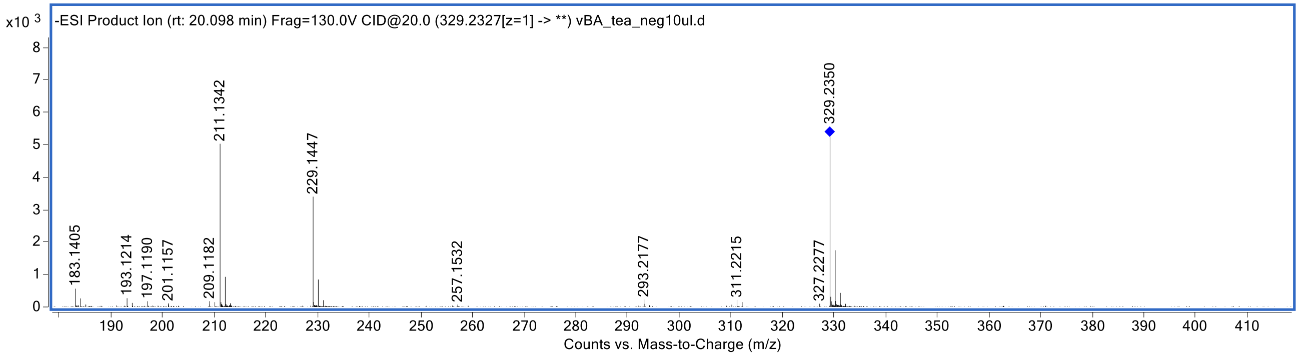

</td></tr>
</table>

<table>
<tr><td>2,3-Dihydroxystearic acid</td><td>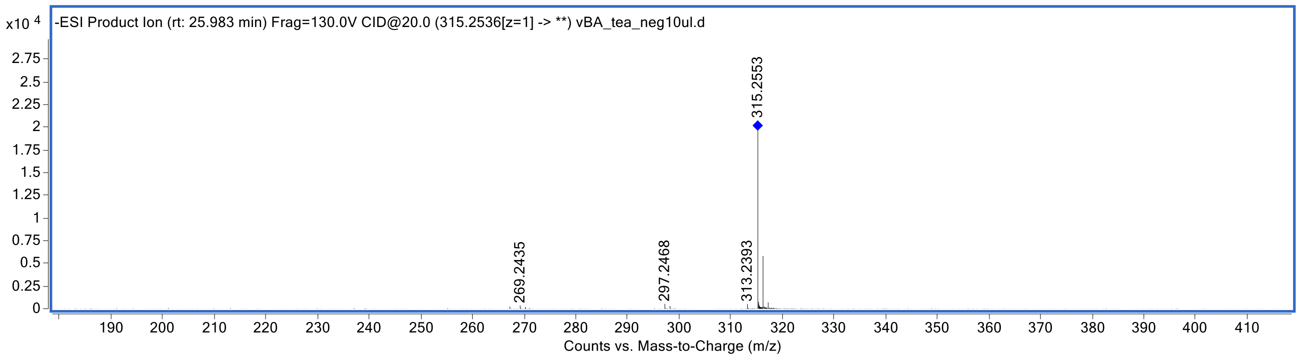
</td></tr>
<tr><td>2-Butyl glucosinolate</td><td>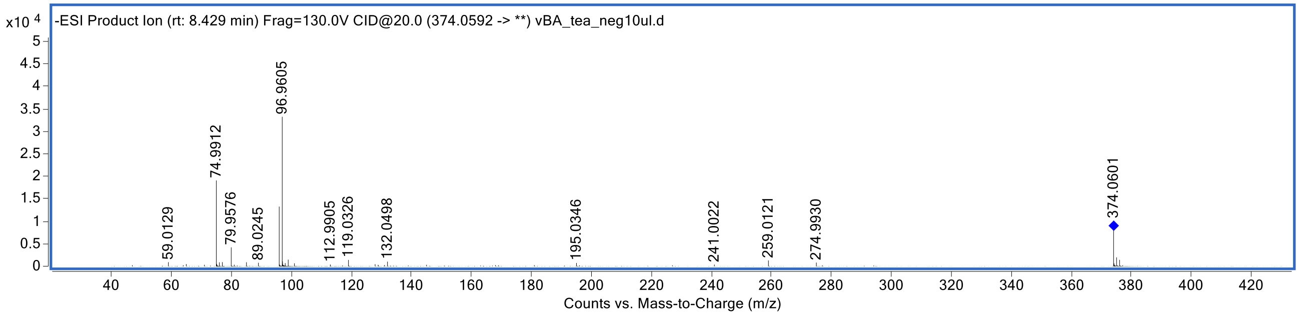
</td></tr>
</table>

3,12-dihydroxypalmitic acid	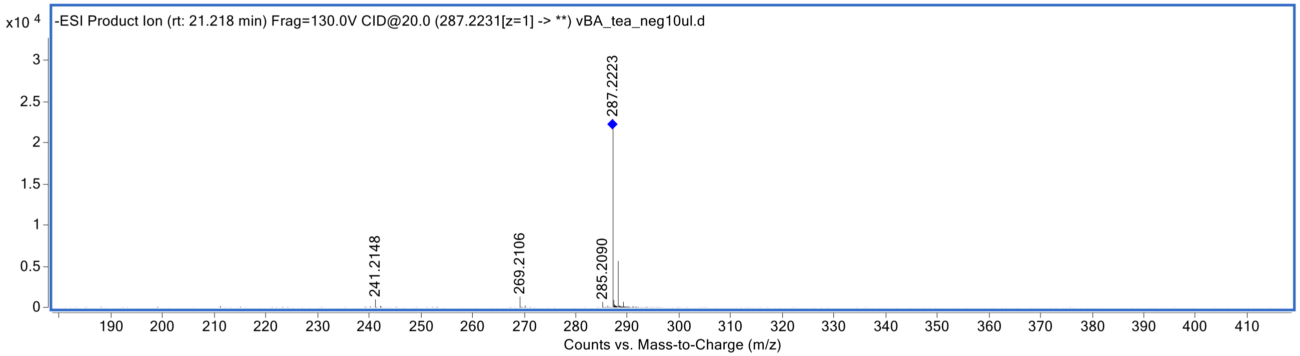
dodecanoic acid	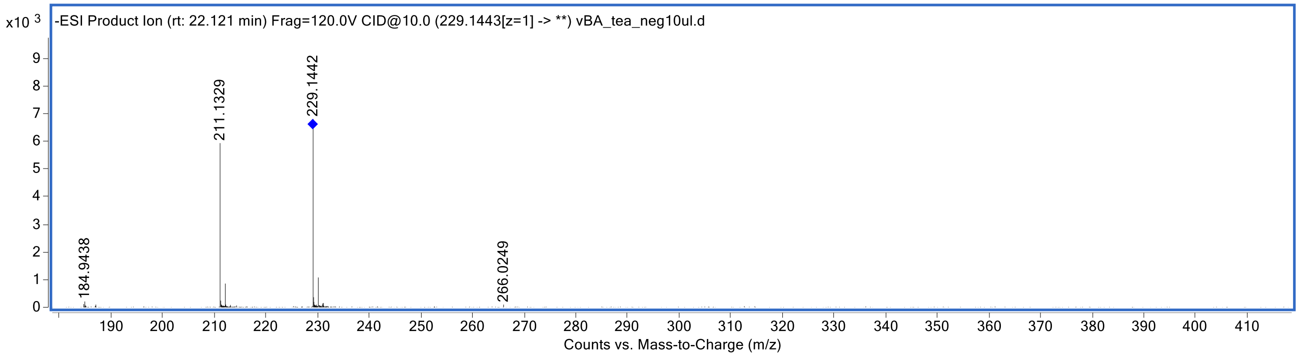

ethyl 3,4-dihydroxybenzoate	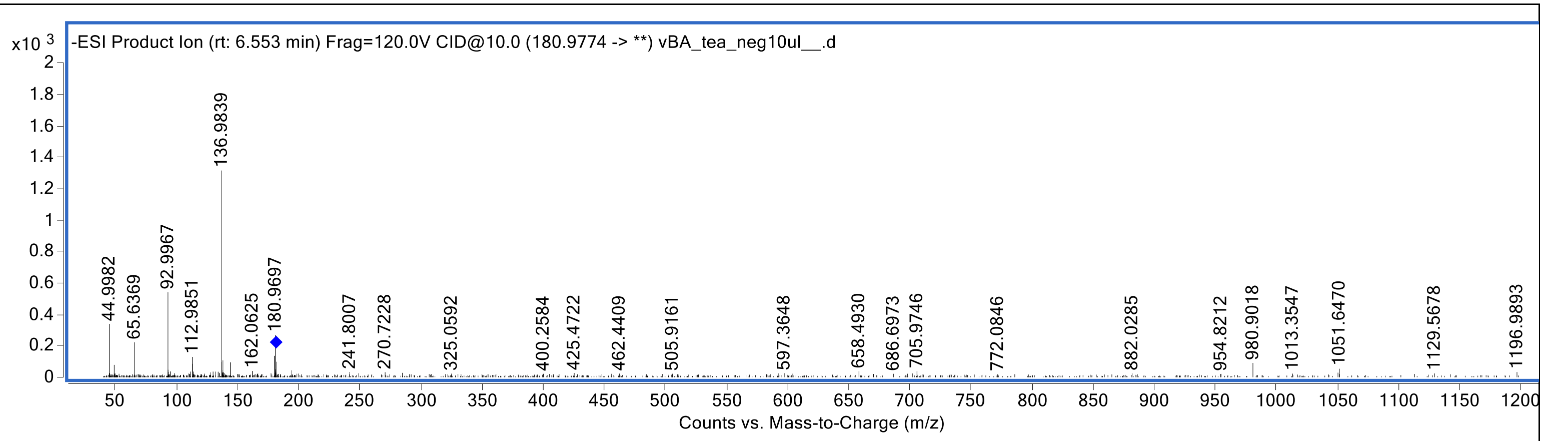
Hexadecanoic acid	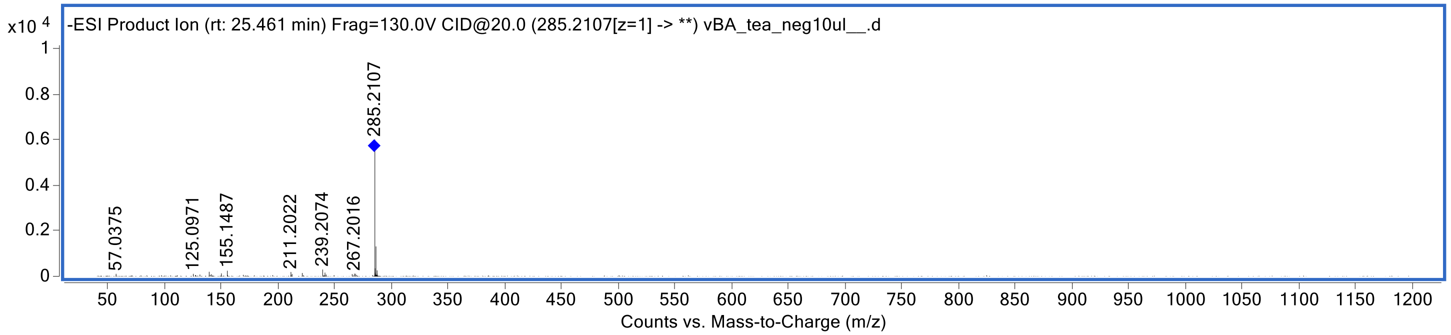

Isopropylglucosinolate	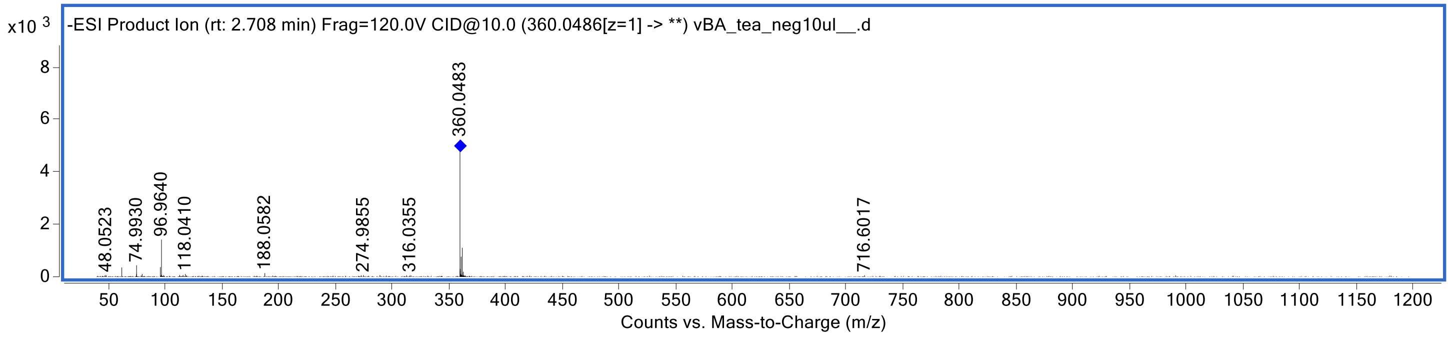
Isorhamnetin rutinoside	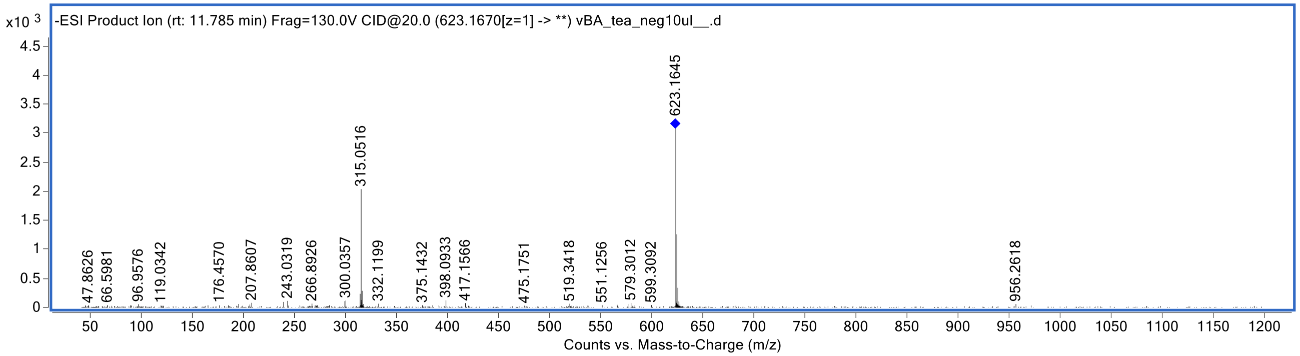

<table>
<tr>
<td>Kaempferol 3-(2G-rhamnosylrutinoside)</td>
<td>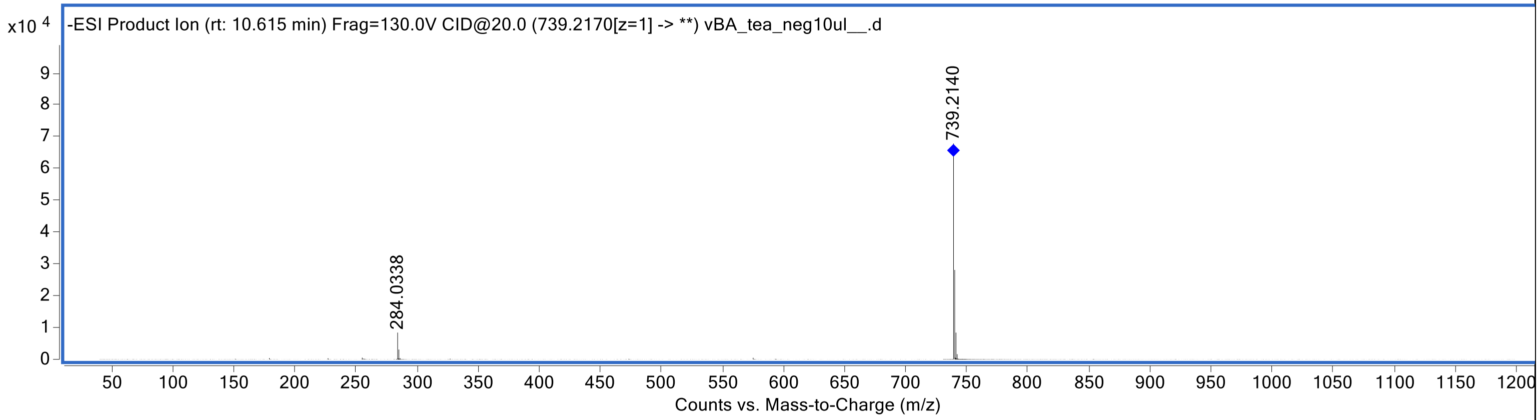
</td>
</tr>
<tr>
<td>Kaempferol 3-neohesperidoside</td>
<td>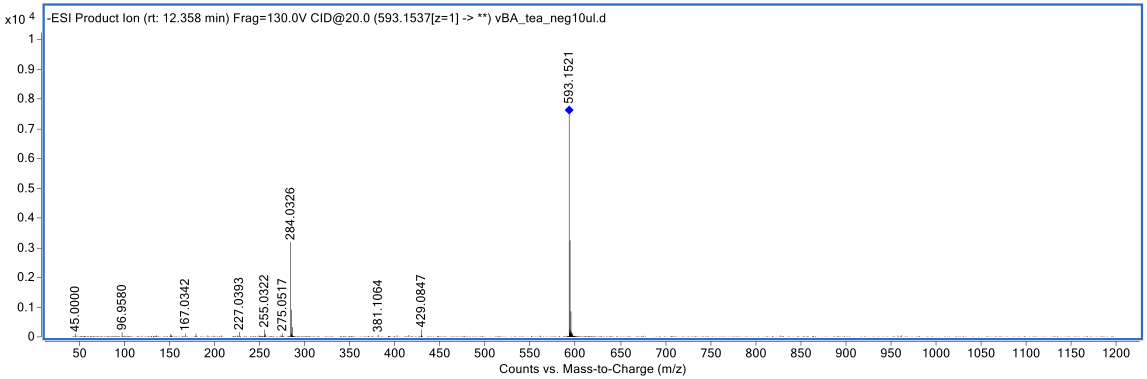
</td>
</tr>
</table>

**Kaempferol-3-O-rutinoside**	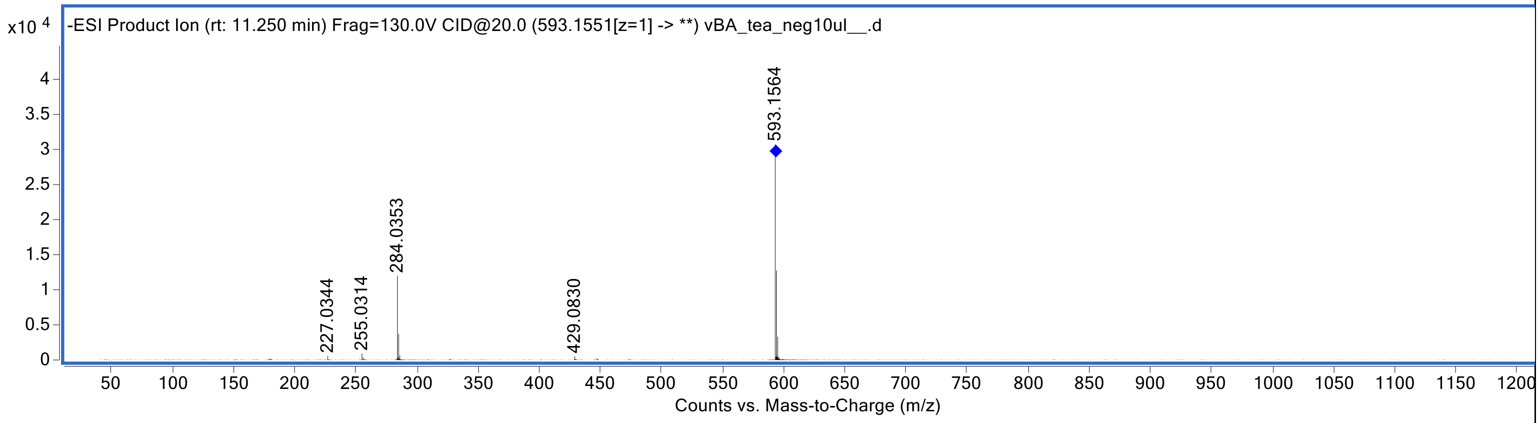
Methyl gallate	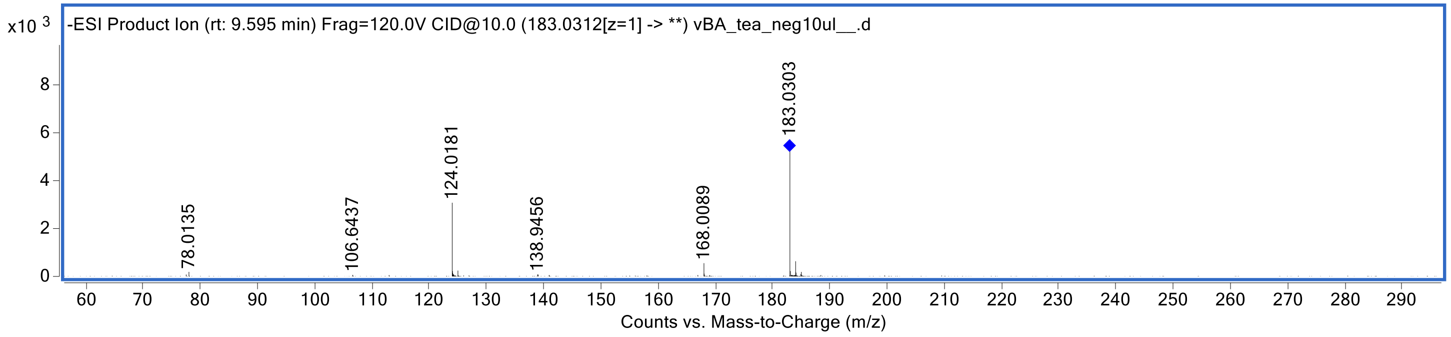

Octadecanoic acid	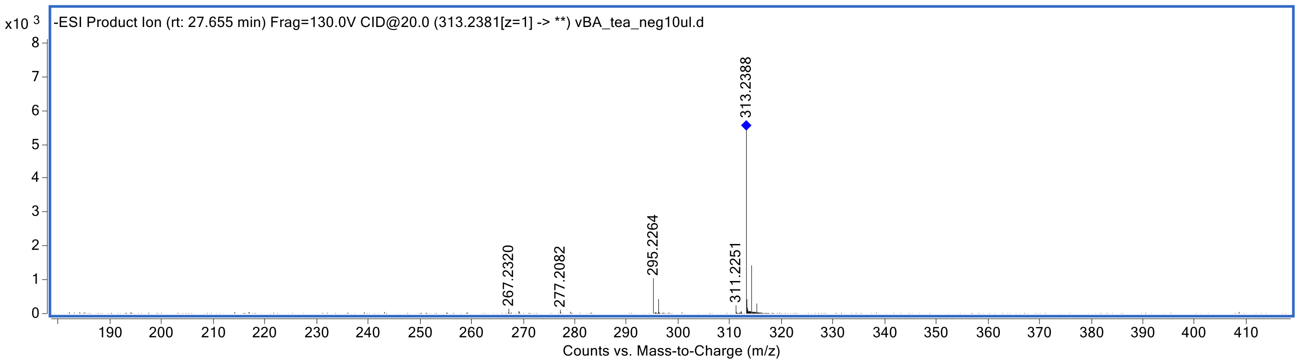

Protocatechuic aldehyde	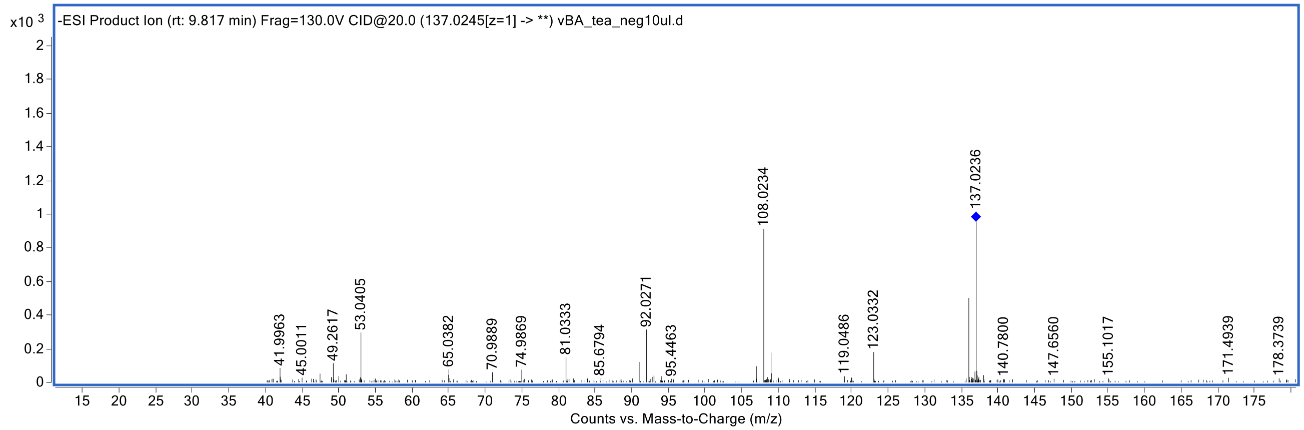
Quercetin 3-(2G-rhamnosylrutinoside	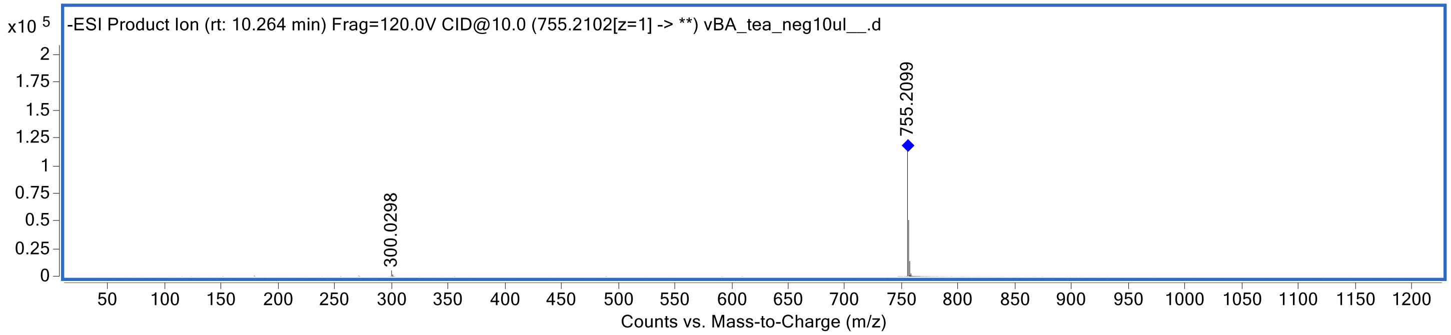

Quercetin rhamninoside	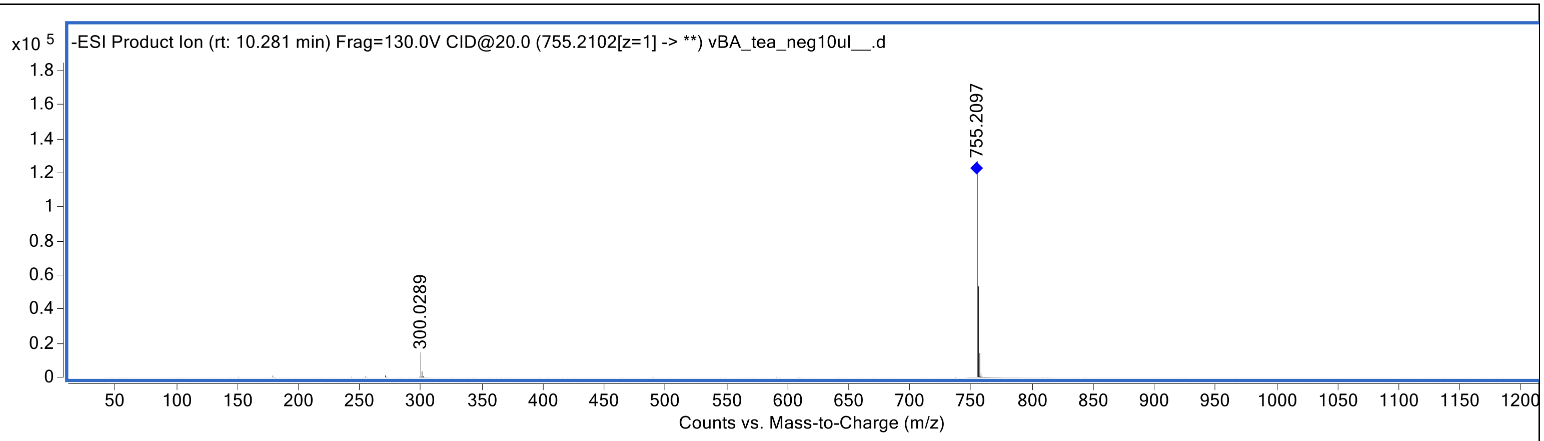
Quercetine neohesperidoside	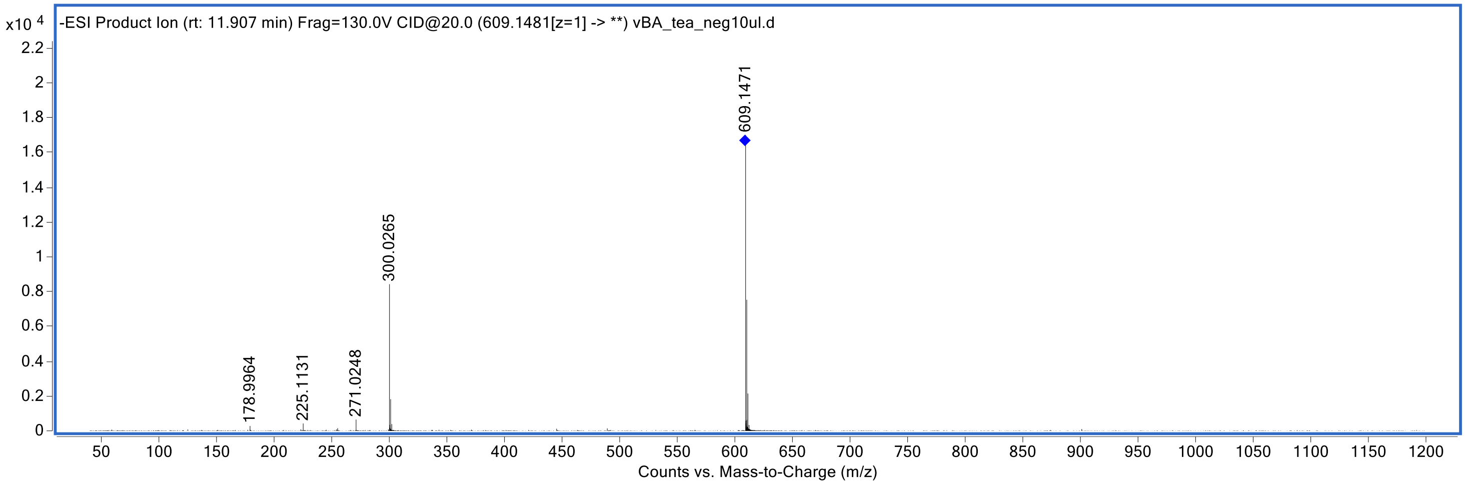

**Racemosin**	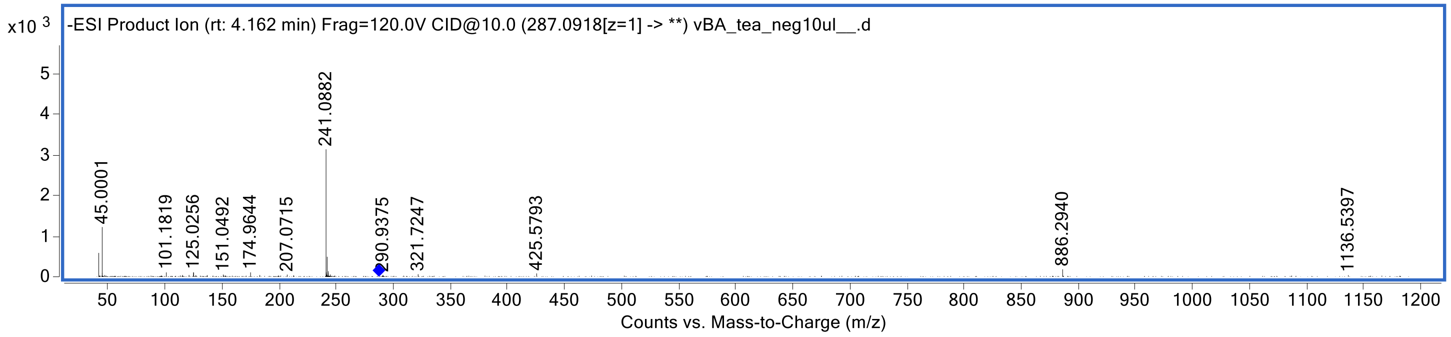
Robinin	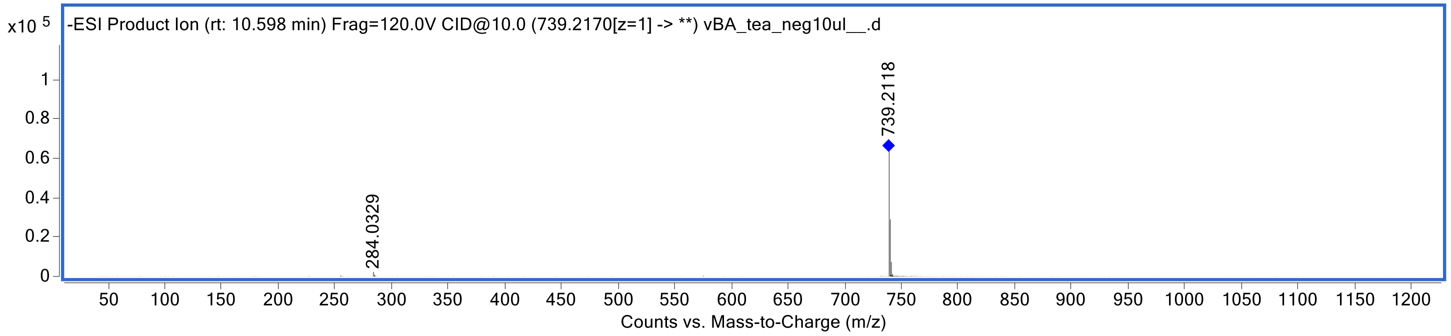

Rutoside	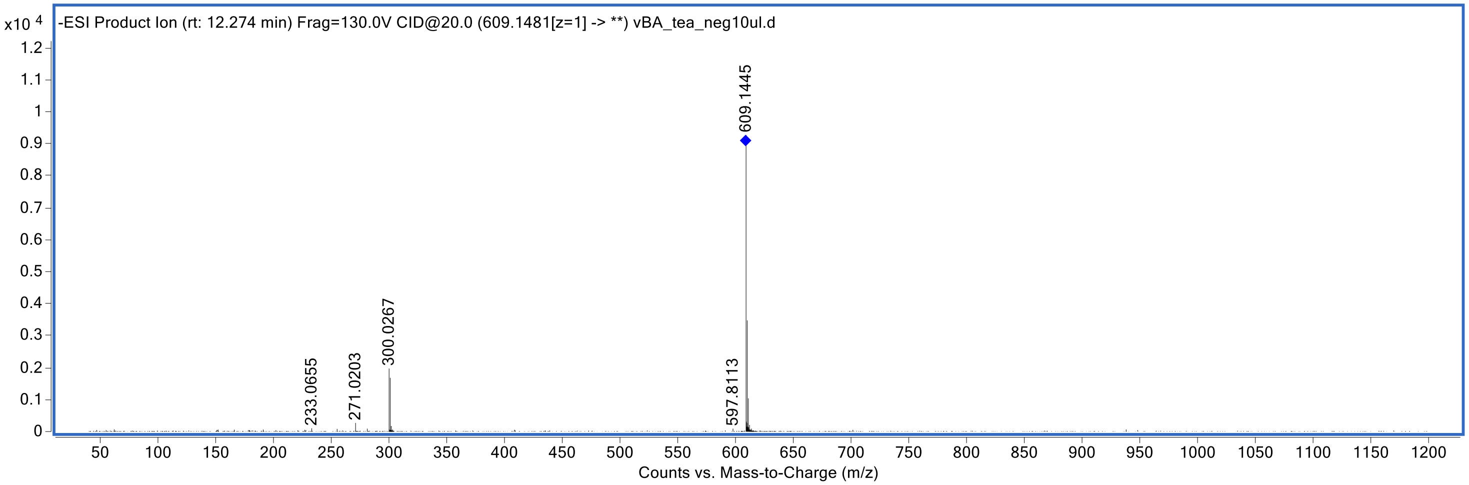

x10 4
-ESI Product Ion (rt: 12.274 min) Frag=130.0V CID@20.0 (609.1481[z=1] -> **) vBA_tea_neg10ul.d
1.2
1.1
1
0.9
0.8
0.7
0.6
0.5
0.4
0.3
0.2
0.1
0
609.1445
300.0267
233.0655
271.0203
597.8113
50 100 150 200 250 300 350 400 450 500 550 600 650 700 750 800 850 900 950 1000 1050 1100 1150 1200
Counts vs. Mass-to-Charge (m/z)

Sacranoside A	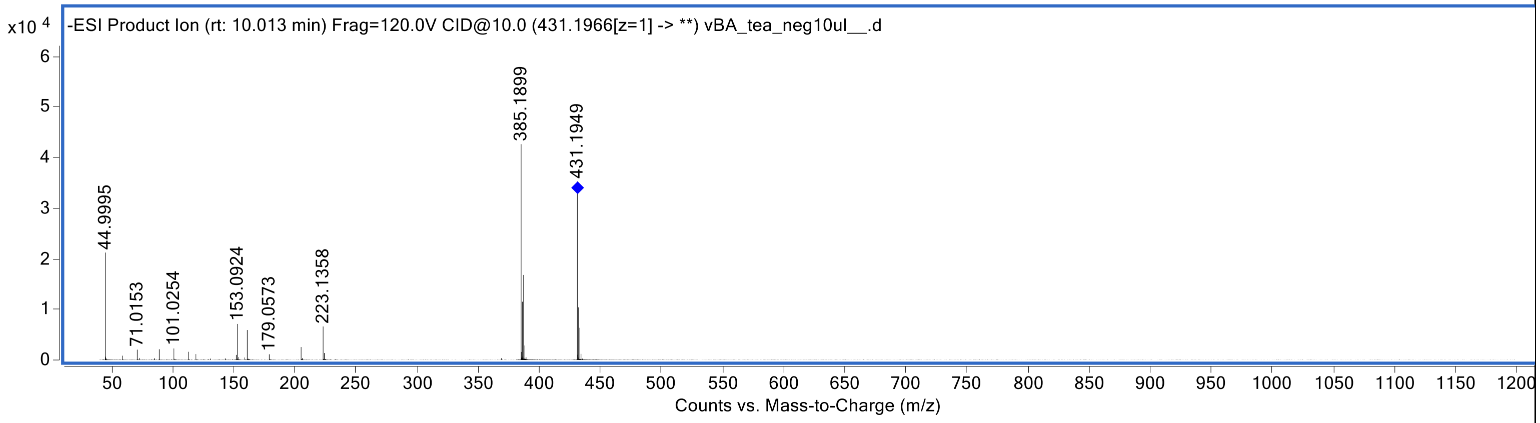
Salicylic acid	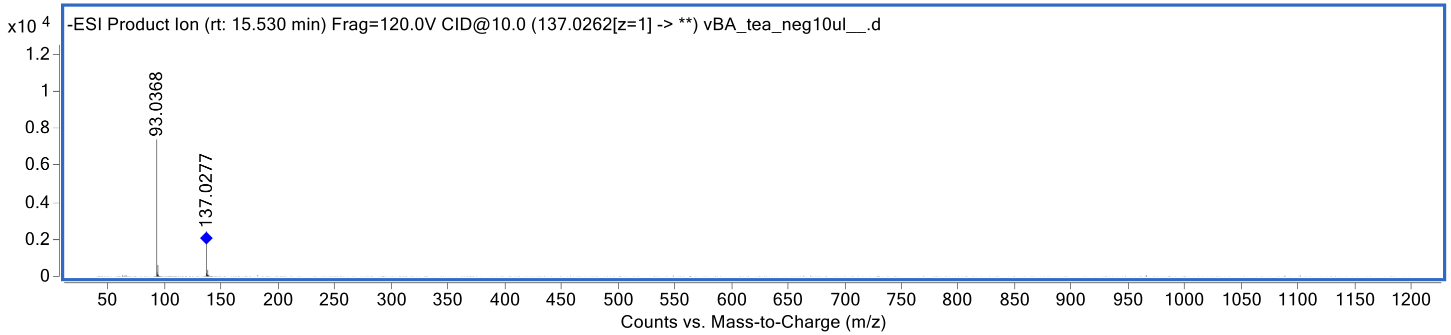

Scortechinone A	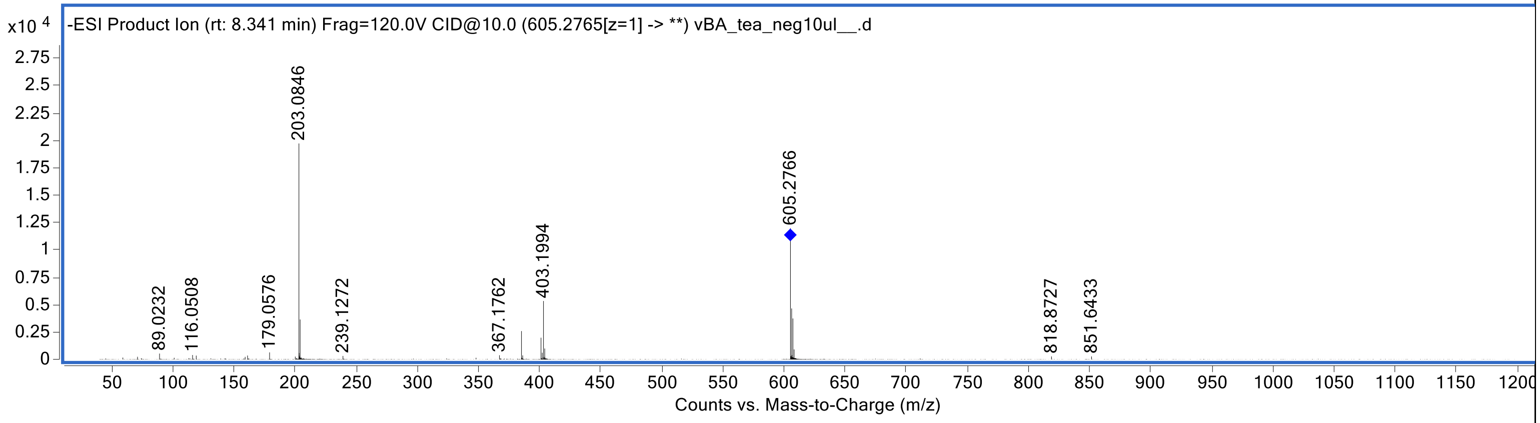
**Isopropylglucosinolate**	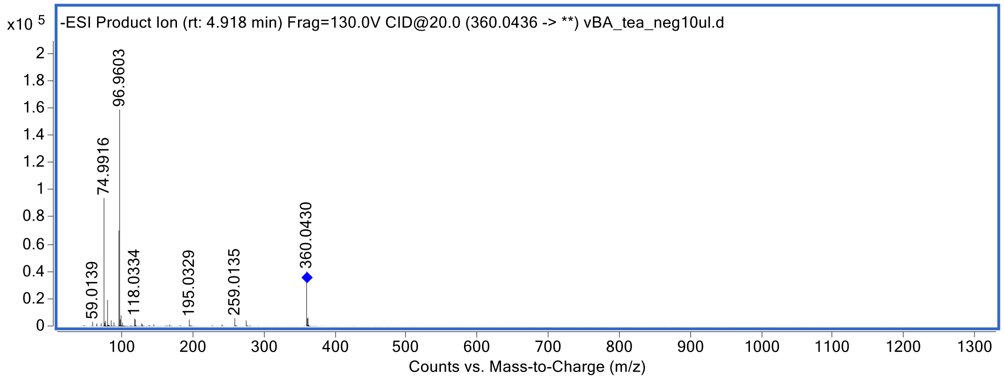

Supplementary Figure 1. **^{1}HNMR and ^{13}CNMR of the compounds isolated from CC.**

<u>Compound 1 (Manghaslin)</u>

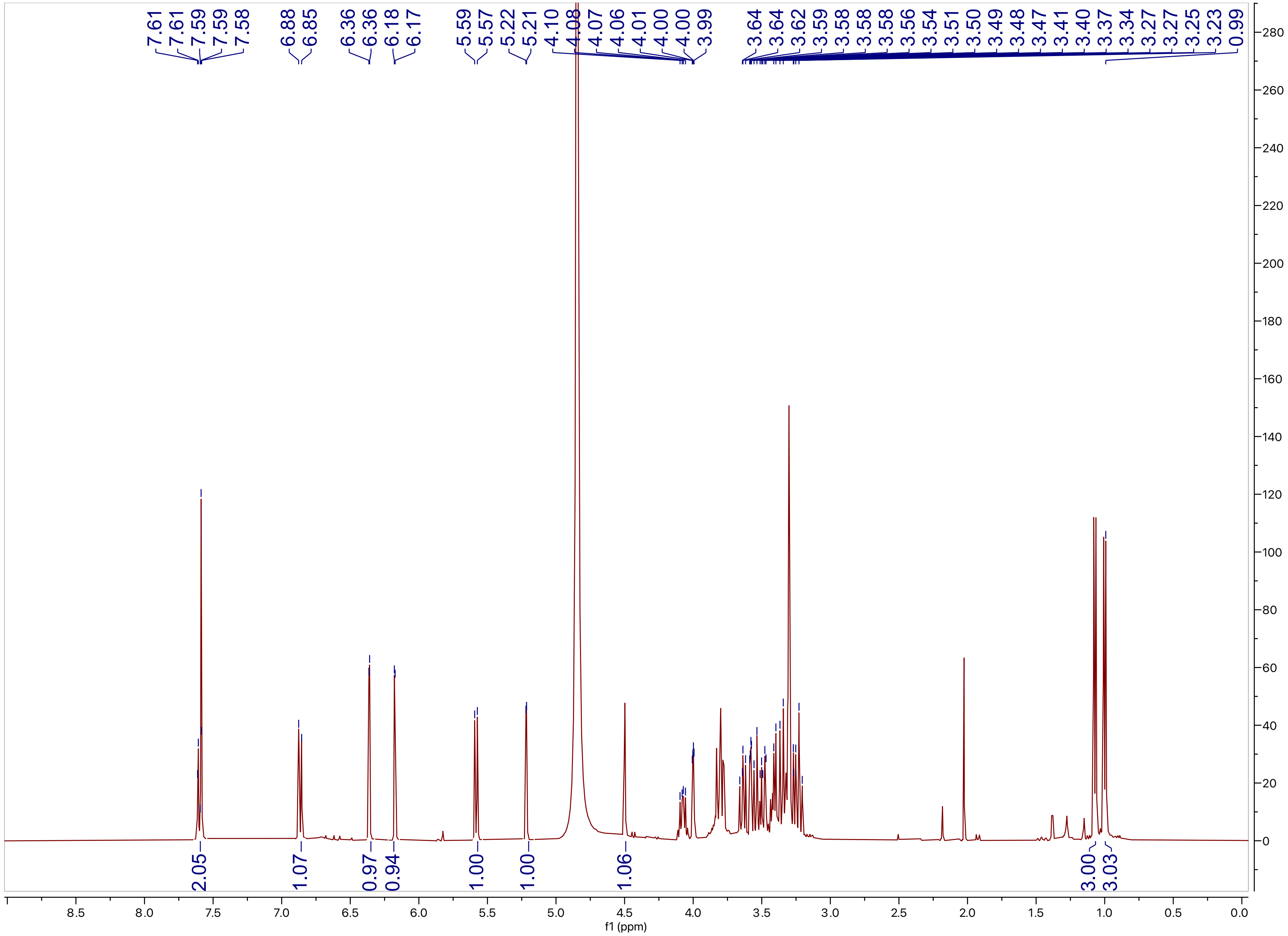

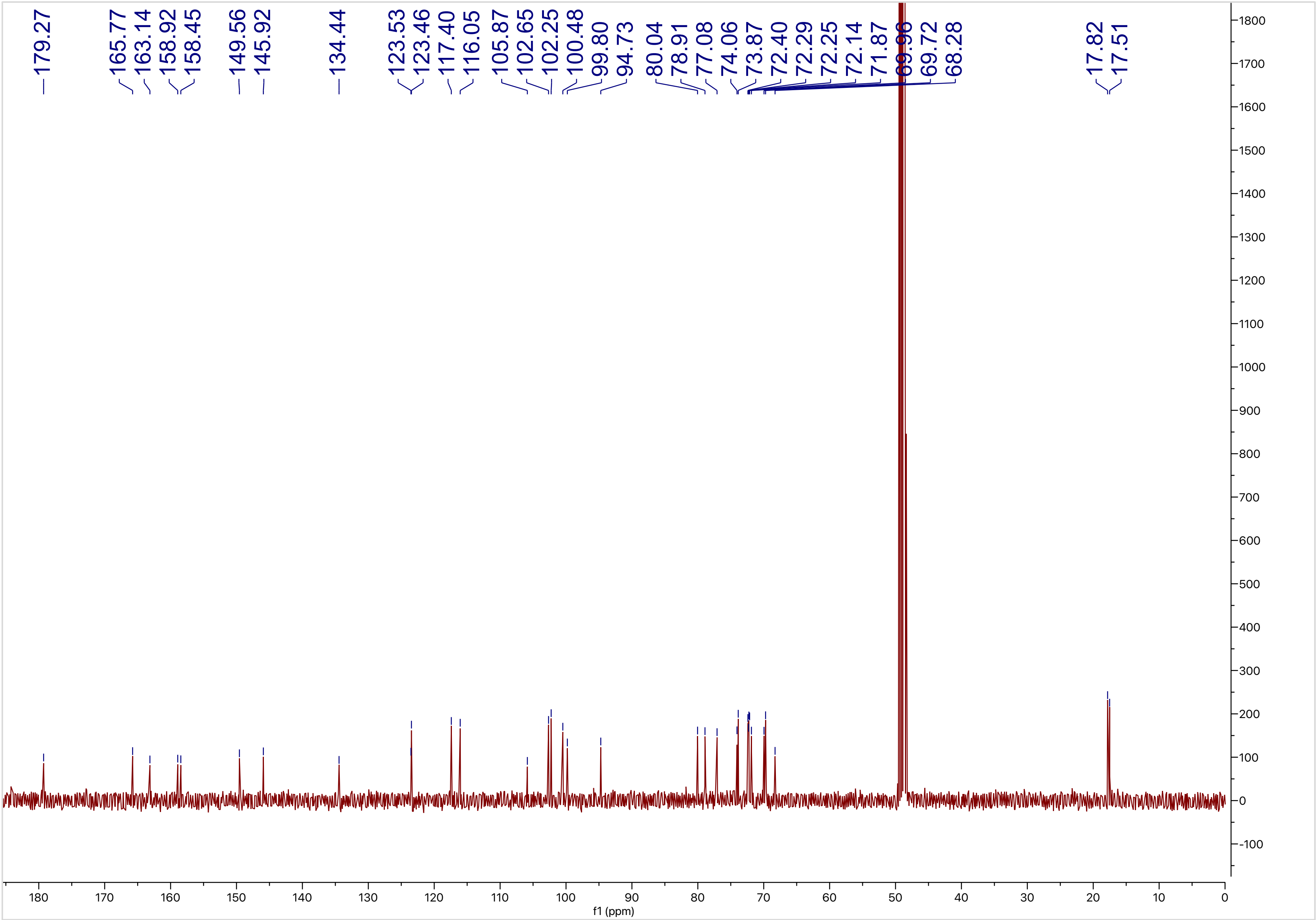
179.27
165.77
163.14
158.92
158.45
149.56
145.92
134.44
123.53
123.46
117.40
116.05
105.87
102.65
102.25
100.48
99.80
94.73
80.04
78.91
77.08
74.06
73.87
72.40
72.29
72.25
72.14
71.87
69.96
69.72
68.28
17.82
17.51
f1 (ppm)

Compound 2 (Clitorin)

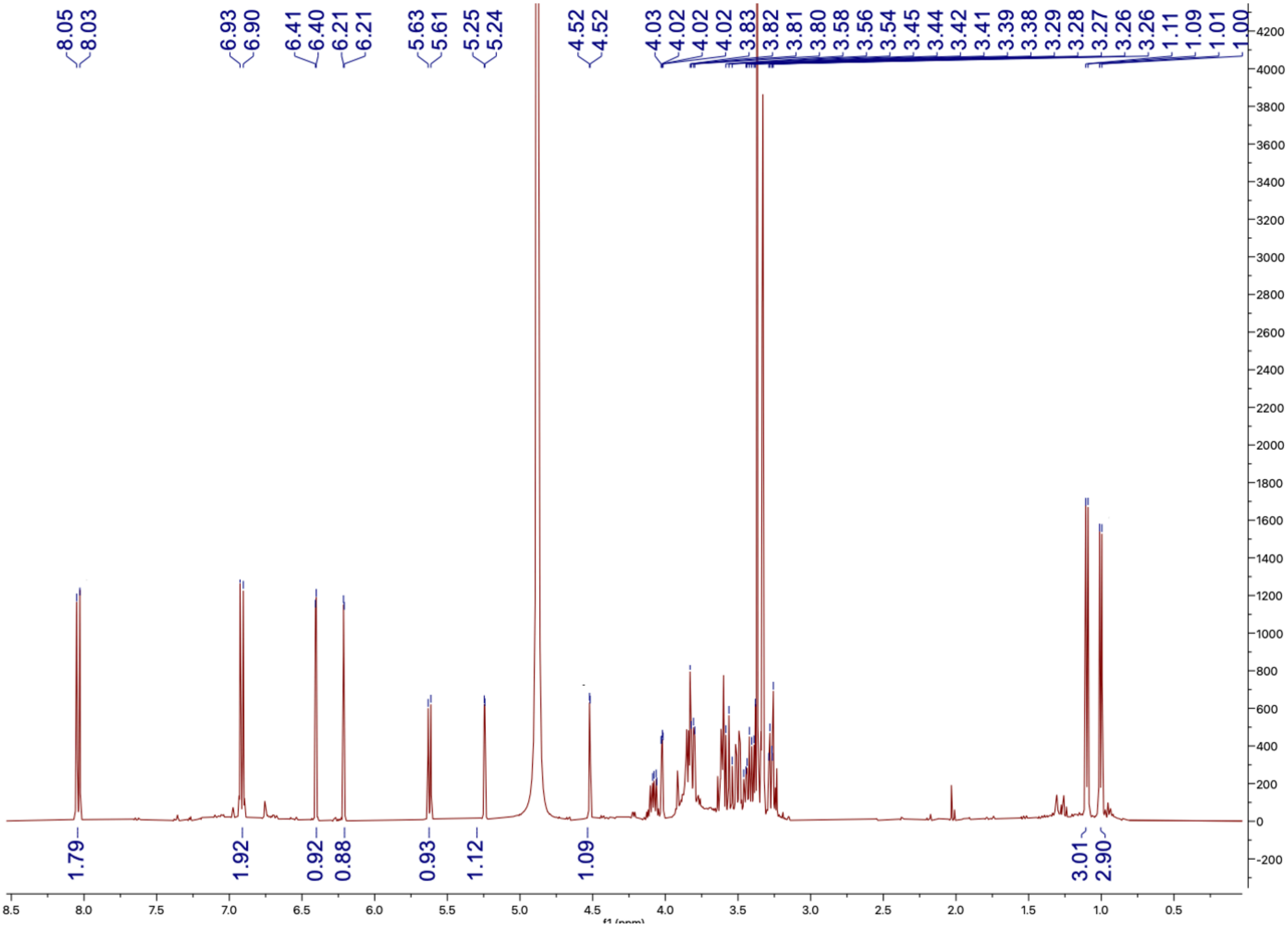

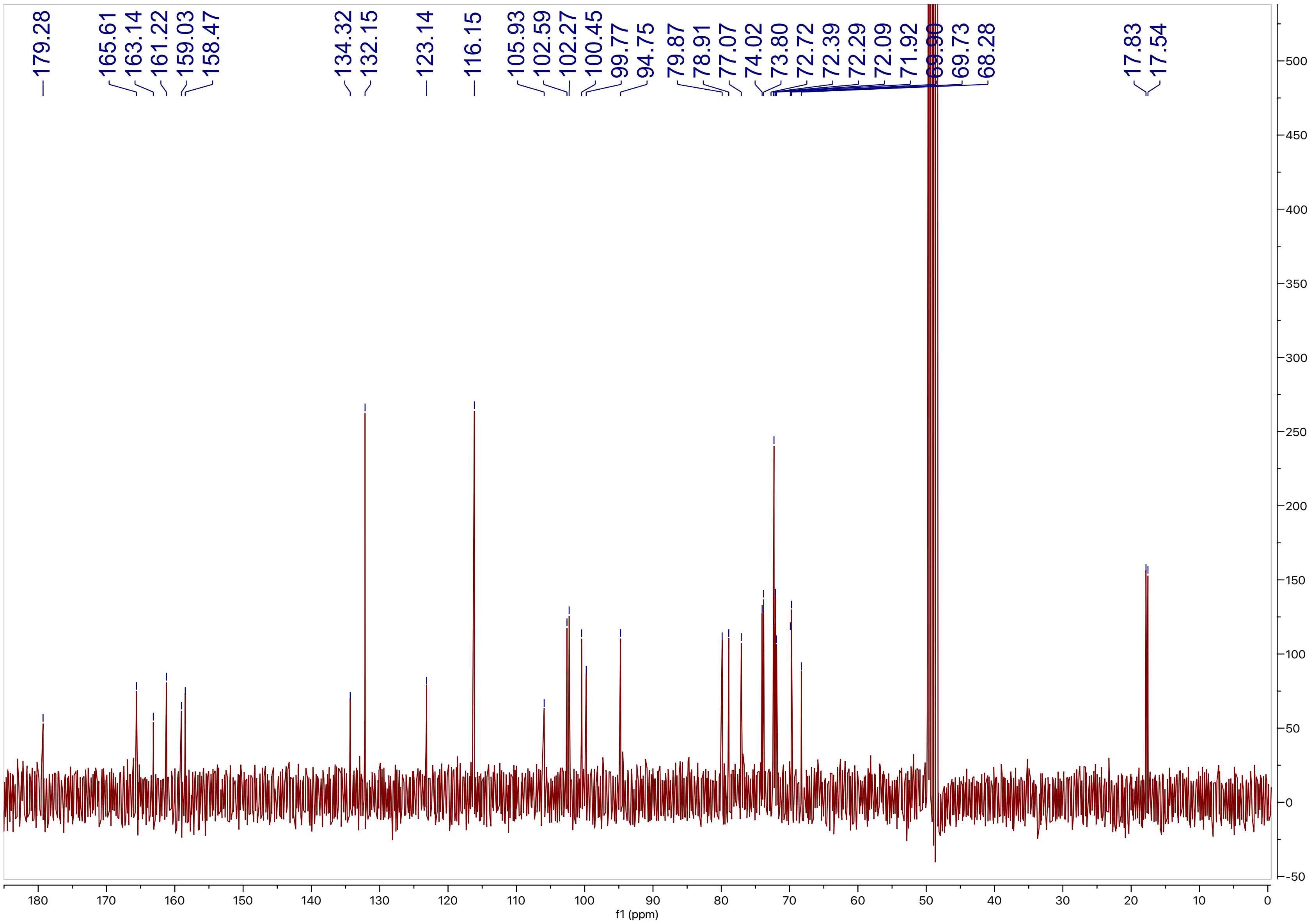
179.28
165.61
163.14
161.22
159.03
158.47
134.32
132.15
123.14
116.15
105.93
102.59
102.27
100.45
99.77
94.75
79.87
78.91
77.07
74.02
73.80
72.72
72.39
72.29
72.09
71.92
69.90
69.73
68.28
17.83
17.54
f1 (ppm)

Compound 3 (Quercetin 3-*O*-neohesperidoside)

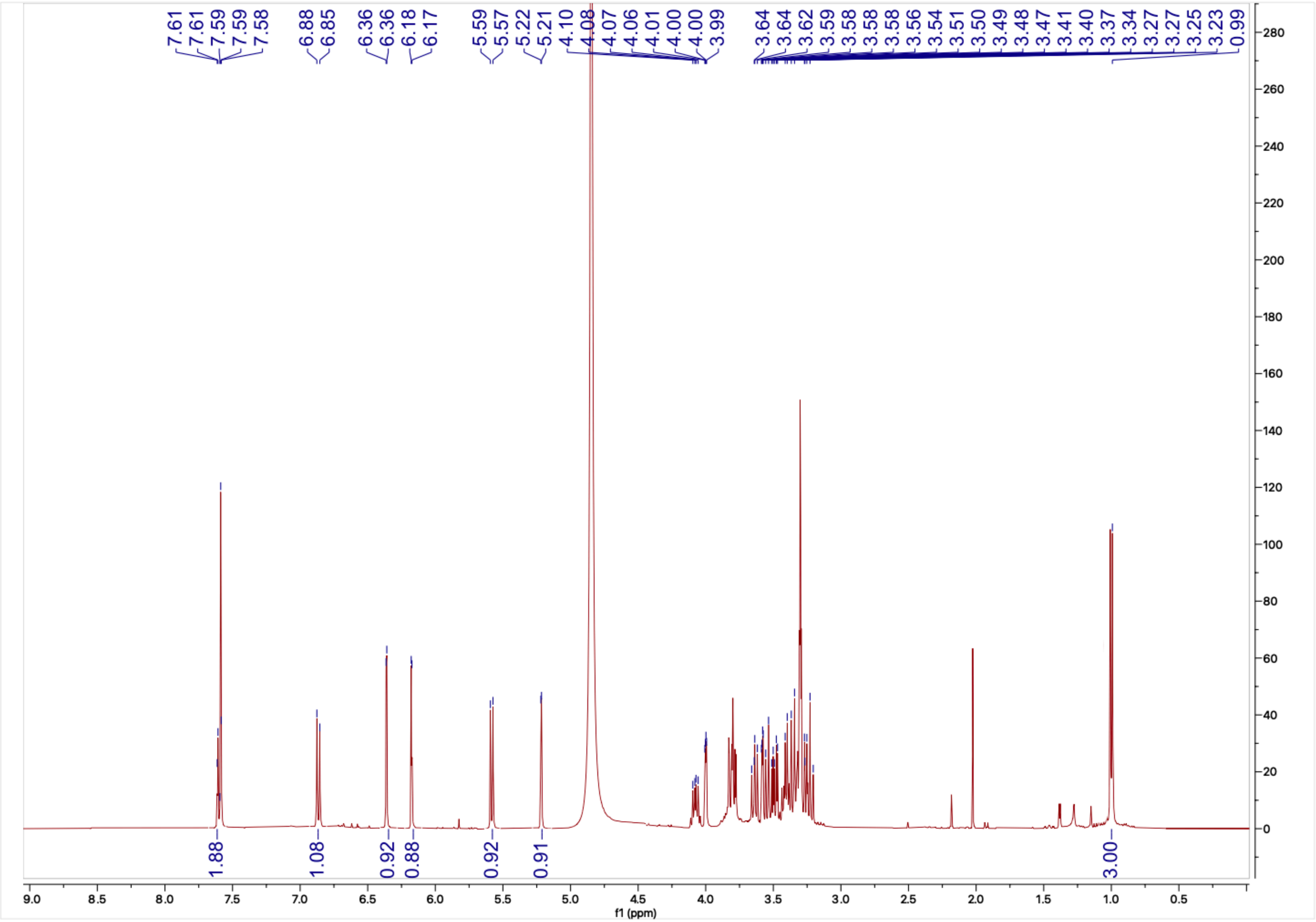

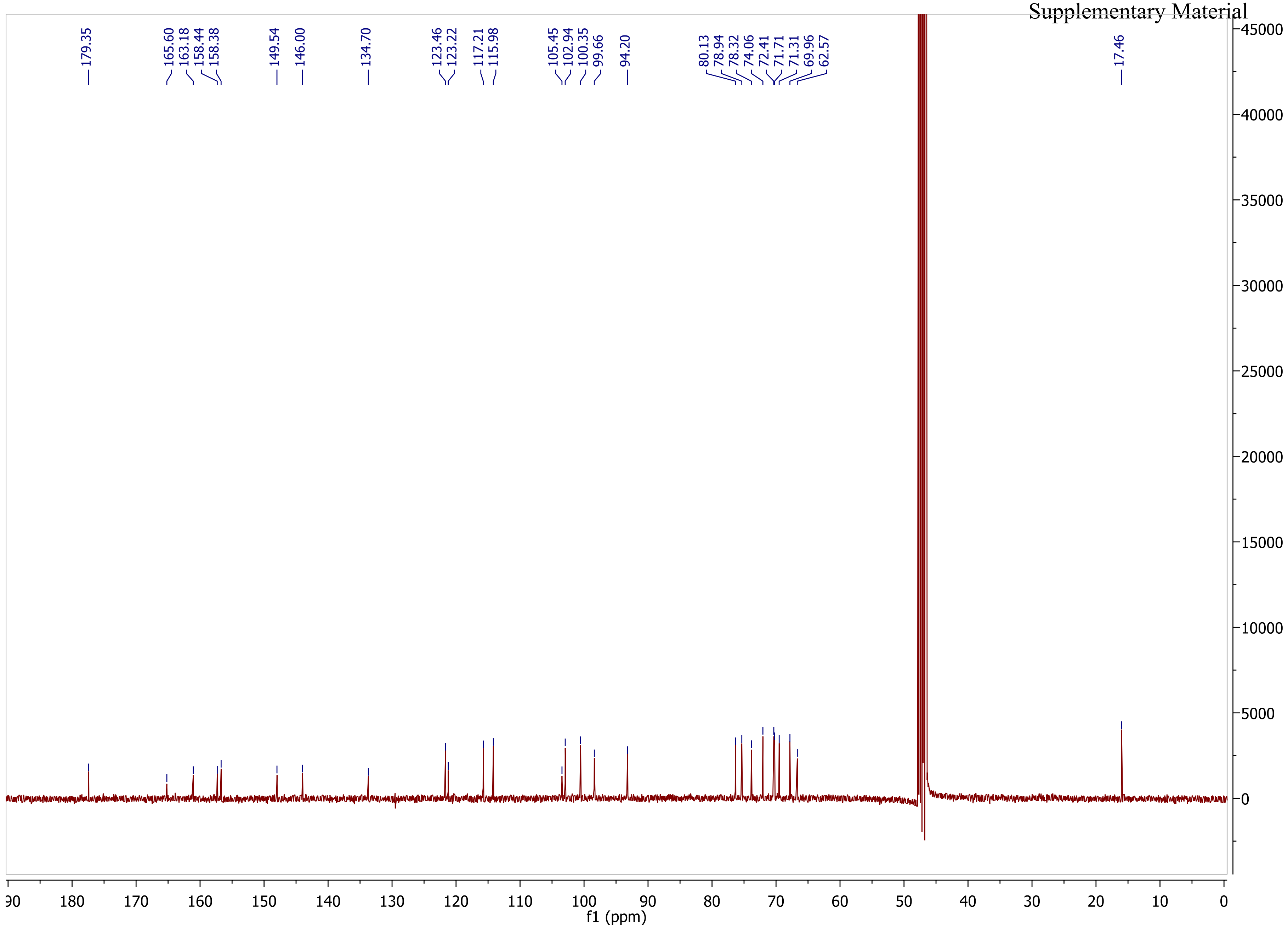
179.35
165.60
163.18
158.44
158.38
149.54
146.00
134.70
123.46
123.22
117.21
115.98
105.45
102.94
100.35
99.66
94.20
80.13
78.94
78.32
74.06
72.41
71.71
71.31
69.96
62.57
17.46
f1 (ppm)

Compound 4 (Rutin)

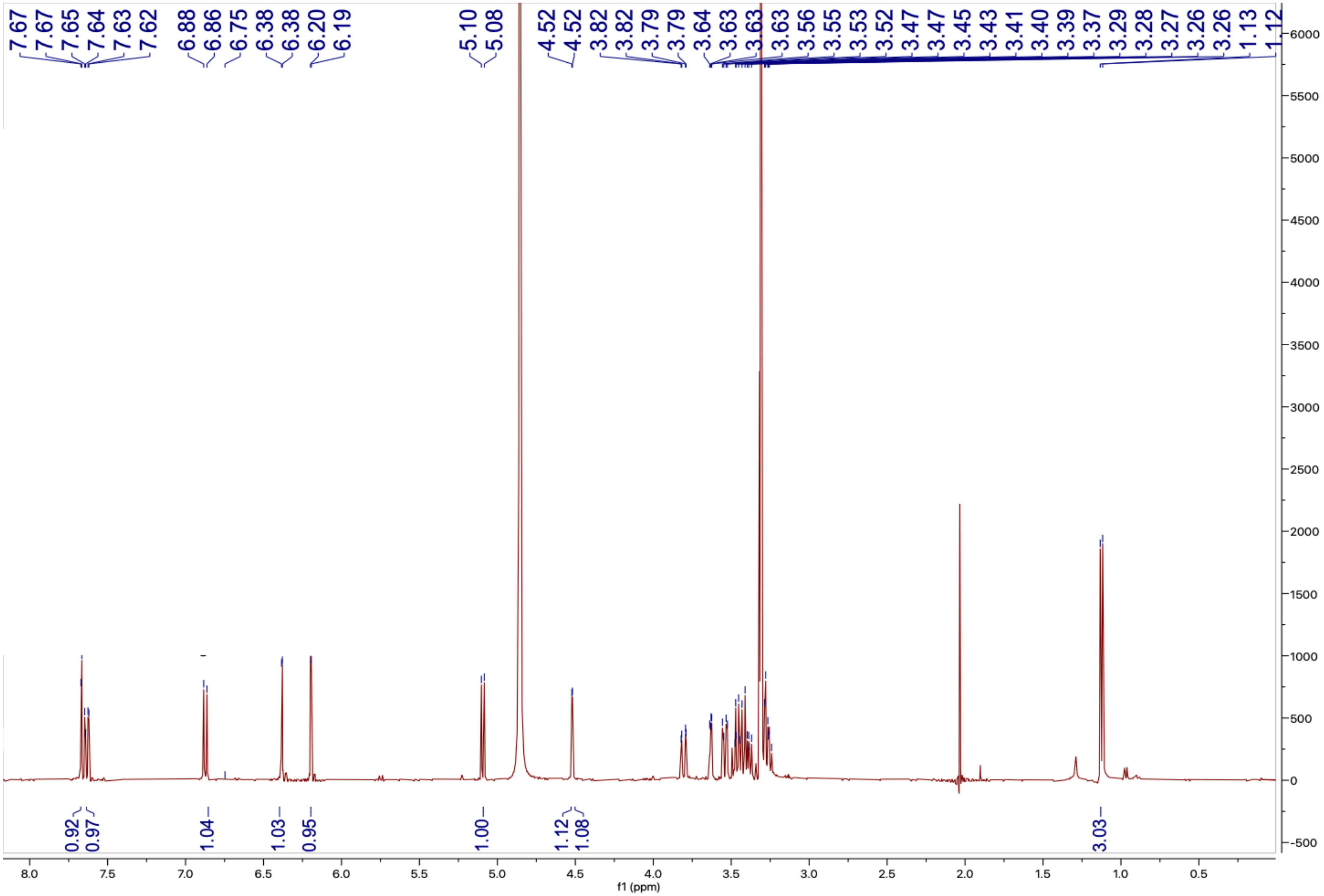

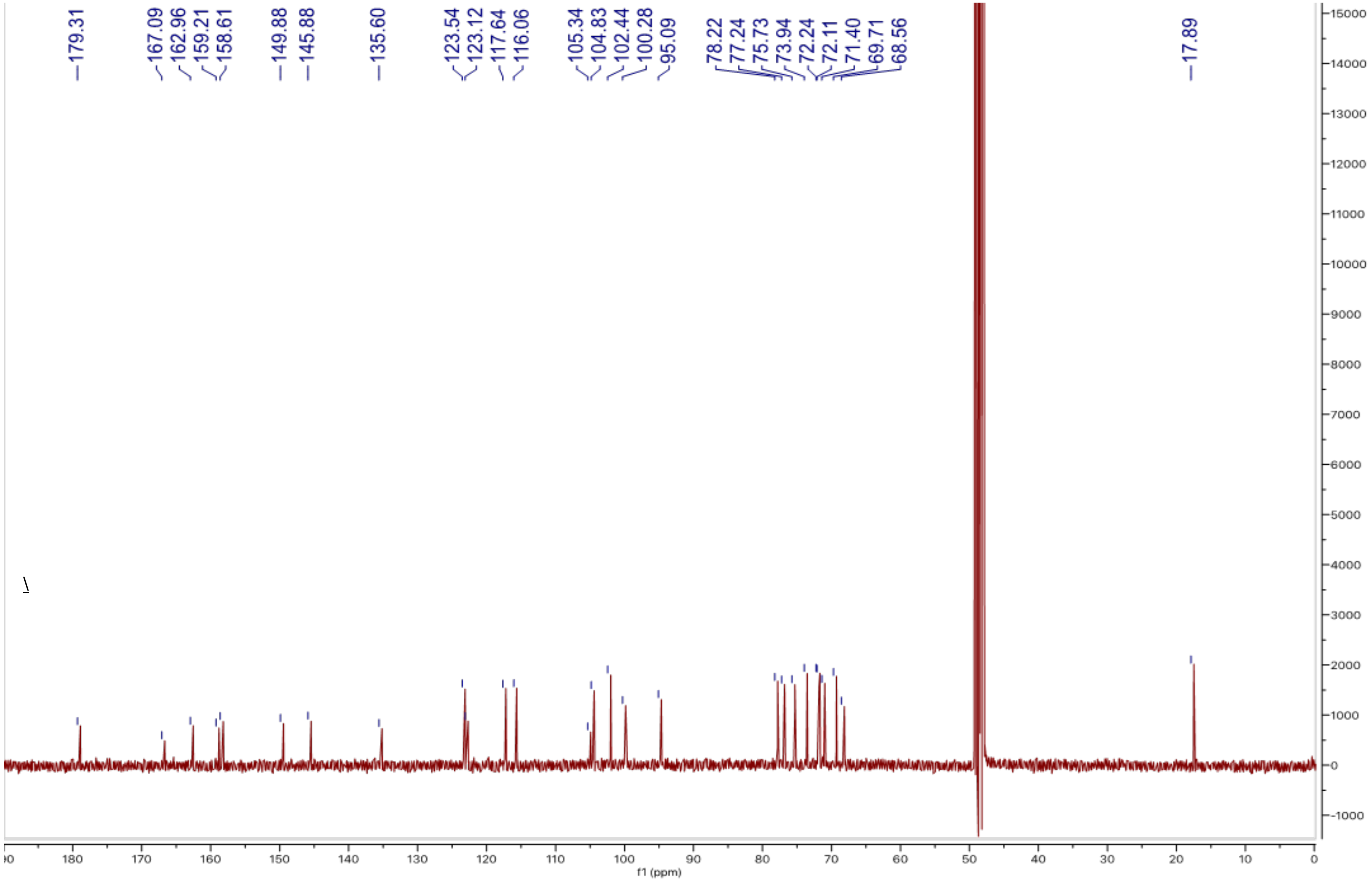
179.31
167.09
162.96
159.21
158.61
149.88
145.88
135.60
123.54
123.12
117.64
116.06
105.34
104.83
102.44
100.28
95.09
78.22
77.24
75.73
73.94
72.24
72.11
71.40
69.71
68.56
17.89
f1 (ppm)

Compound 5 (Kaempferol 3-*O*-neohesperidoside)

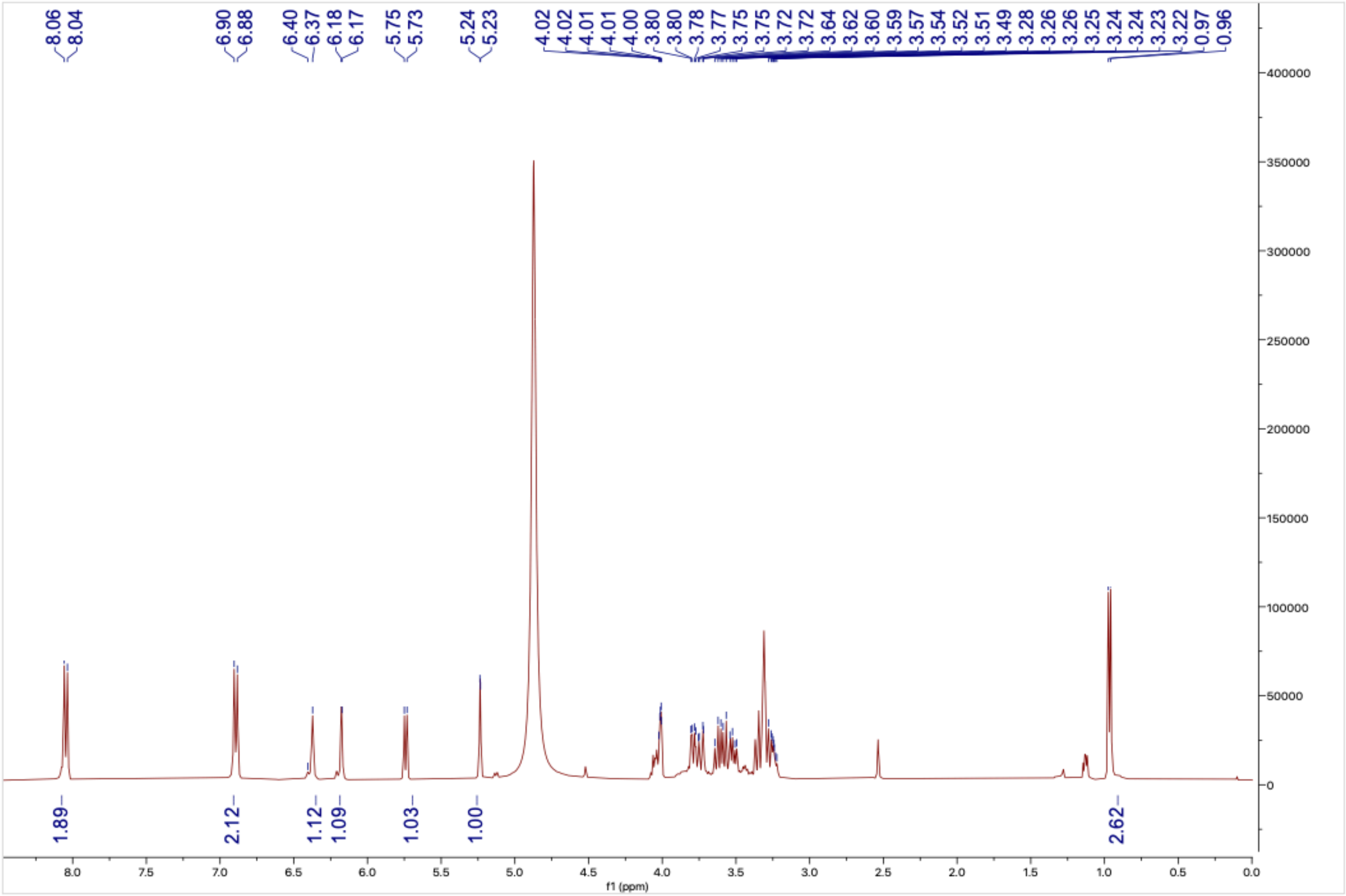

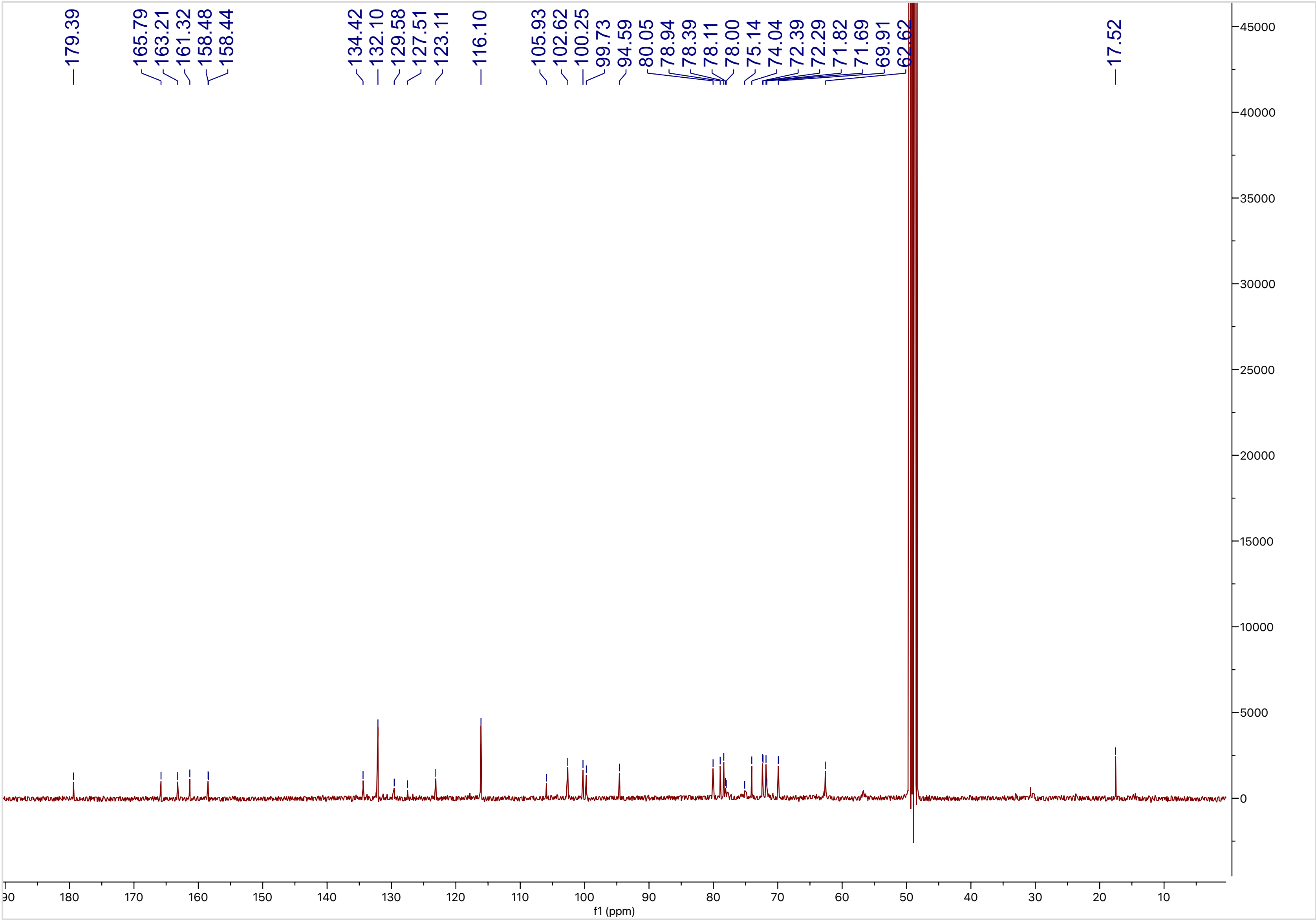
179.39
165.79
163.21
161.32
158.48
158.44
134.42
132.10
129.58
127.51
123.11
116.10
105.93
102.62
100.25
99.73
94.59
80.05
78.94
78.39
78.11
78.00
75.14
74.04
72.39
72.29
71.82
71.69
69.91
62.62
17.52
f1 (ppm)

Compound 6 (Nicotiflorin)

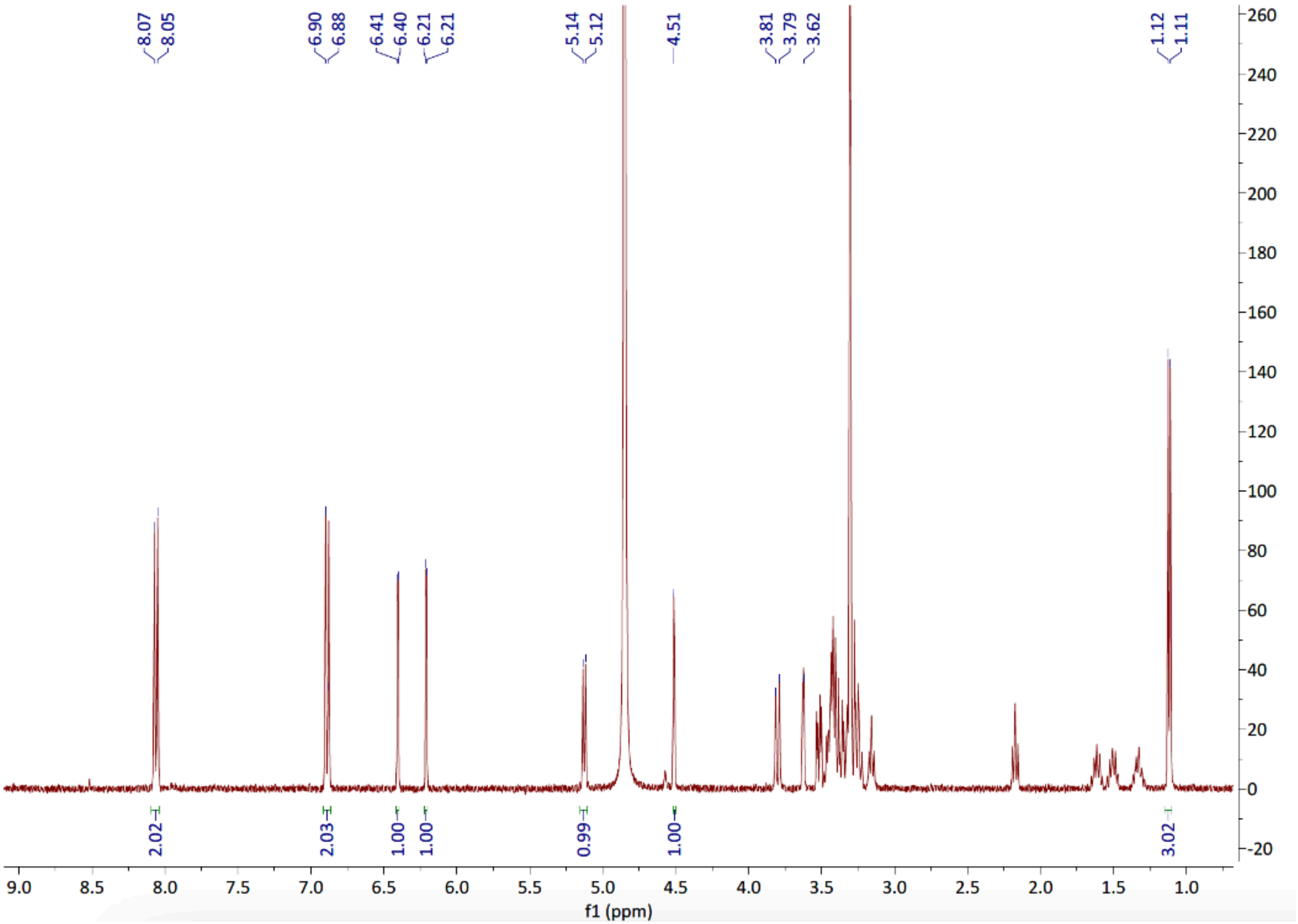

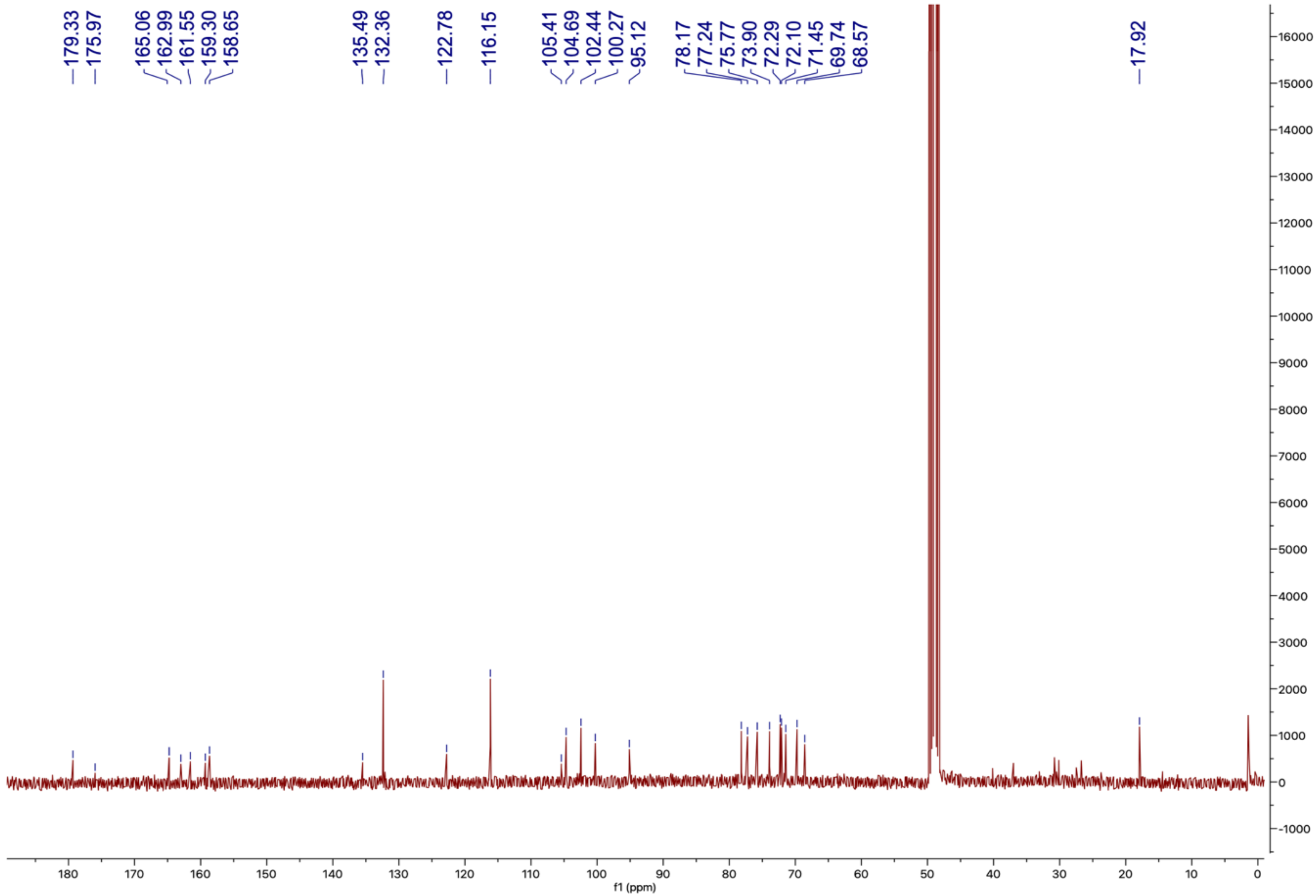
179.33
175.97
165.06
162.99
161.55
159.30
158.65
135.49
132.36
122.78
116.15
105.41
104.69
102.44
100.27
95.12
78.17
77.24
75.77
73.90
72.29
72.10
71.45
69.74
68.57
17.92
f1 (ppm)

Compound 7 (Narcissin)

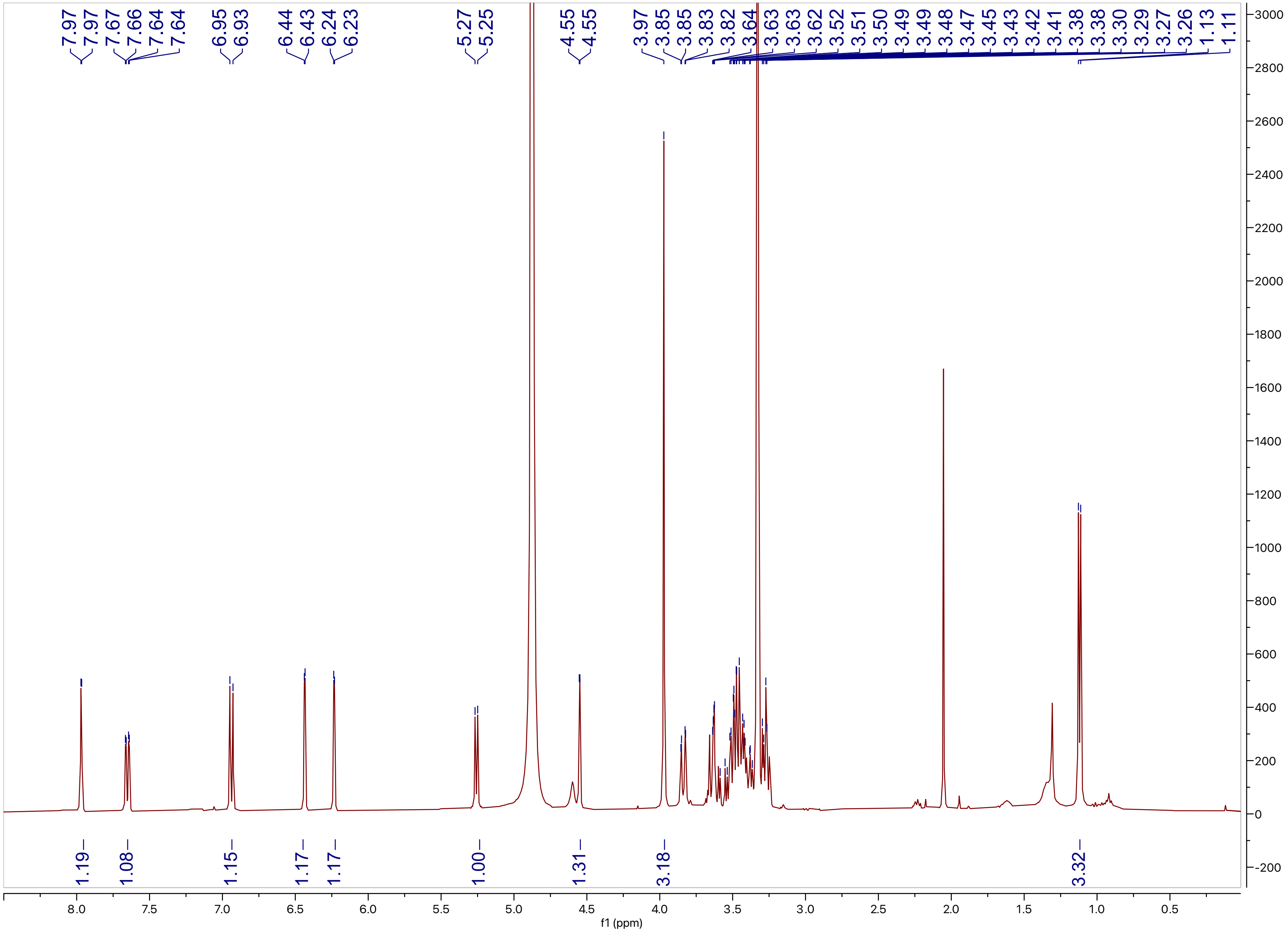

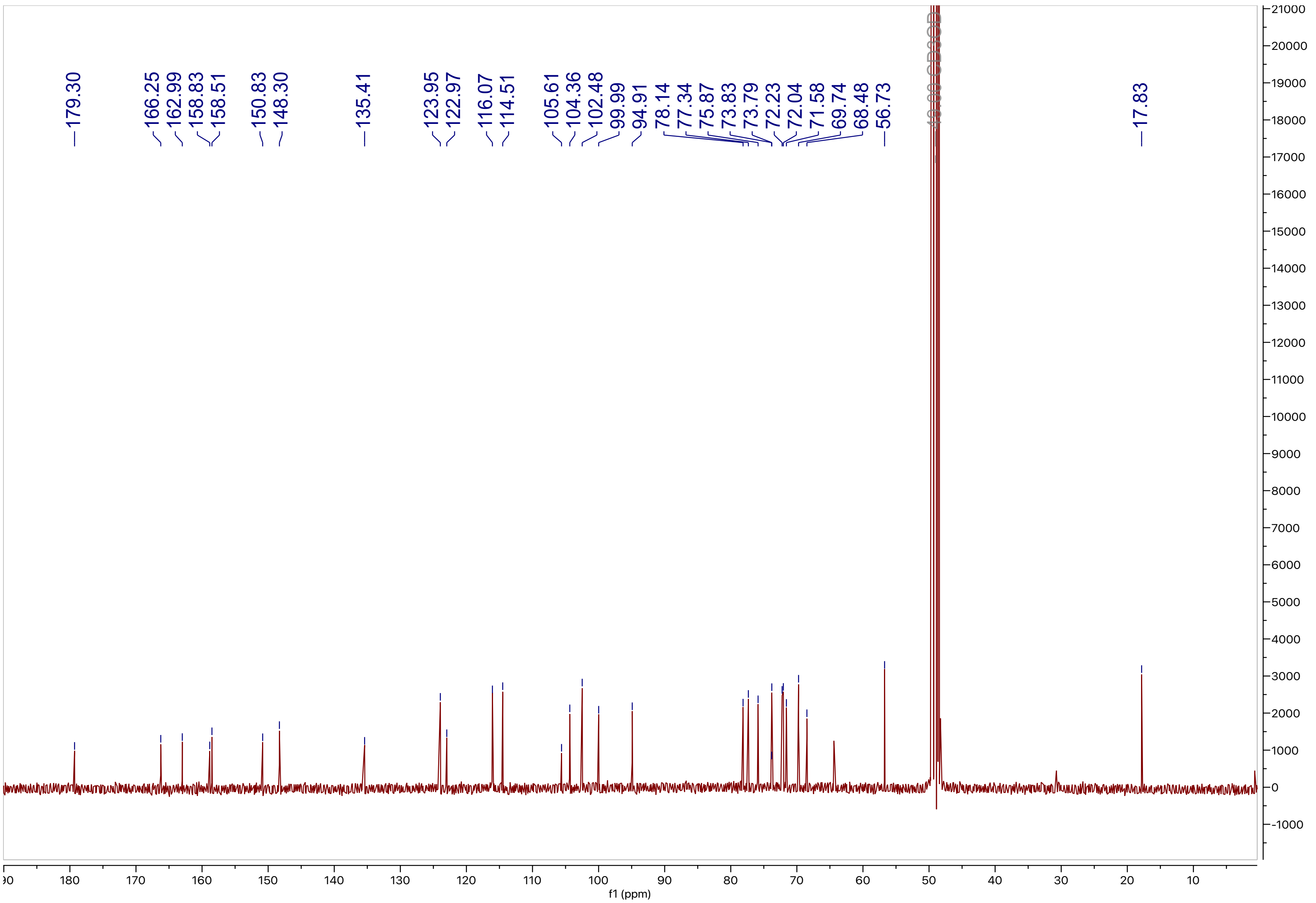
179.30
166.25
162.99
158.83
158.51
150.83
148.30
135.41
123.95
122.97
116.07
114.51
105.61
104.36
102.48
99.99
94.91
78.14
77.34
75.87
73.83
73.79
72.23
72.04
71.58
69.74
68.48
56.73
17.83
f1 (ppm)

Supplementary Figure 2. Structure of MMP-9 inhibitor (5-(4-phenoxyphenyl)-5-(4-pyrimidin-2- ylpiperazin-1-yl) pyrimidine-2,4,6(2h,3h)-trione)). a) 2D inhibitor structure and b) 3D overlay of the inhibitor in its co-crystallized state (blue) and the best Redocking pose (yellow).

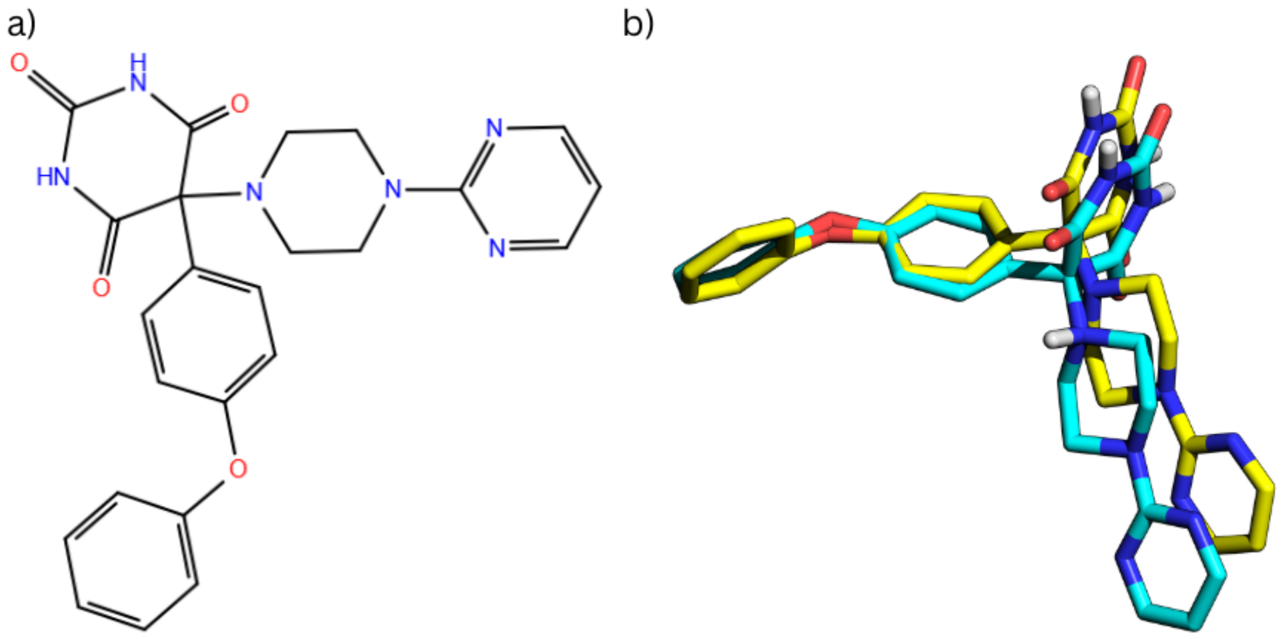

Supplementary Figure 3. Overview of the molecular docking site on the protein binding site. a) Re-docking of the inhibitor (co-crystallized state - blue; best Redocking pose - yellow). b) Docking of seven flavonoids in the MMP-9 protein binding site.

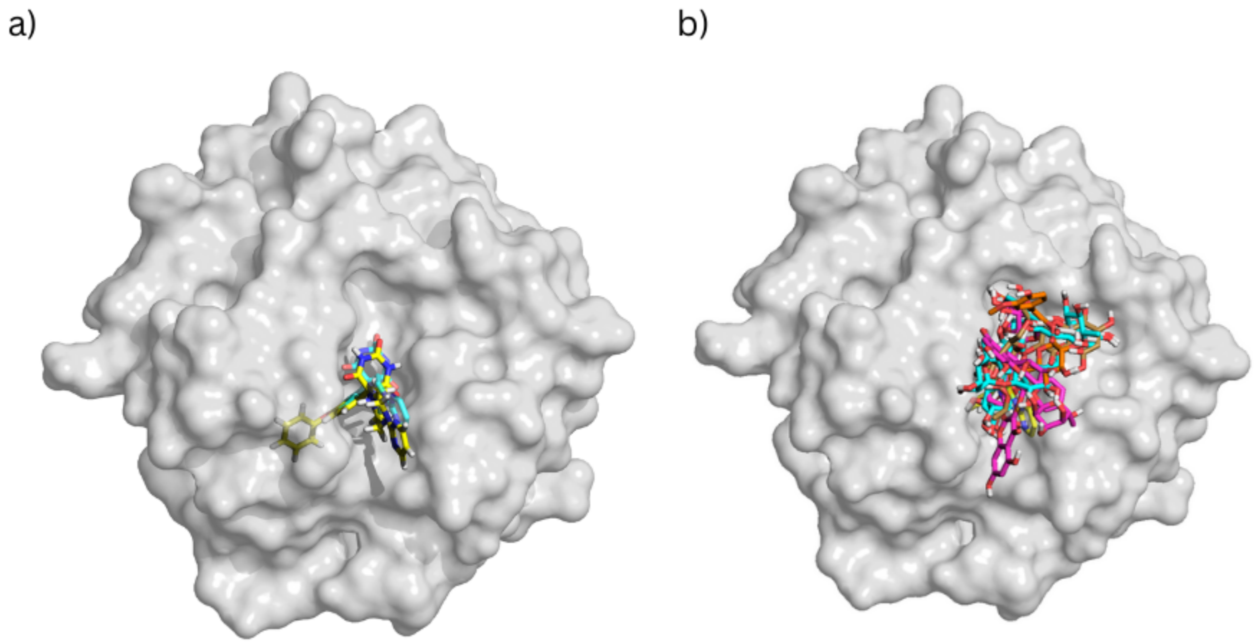

Supplementary Figure 4. Result of molecular docking of the best pose of each flavonoid at the protein interaction site. Amino acids involved in the interaction between protein and ligands. Images obtained from LigPlot+. Purple lines: ligand bonds. Orange lines: amino acids bonds. Green dotted lines: H bonds distances. Red half circle: amino acids involved in hydrophobic interactions. Black dots with red lines: corresponding atoms involved in hydrophobic contacts.

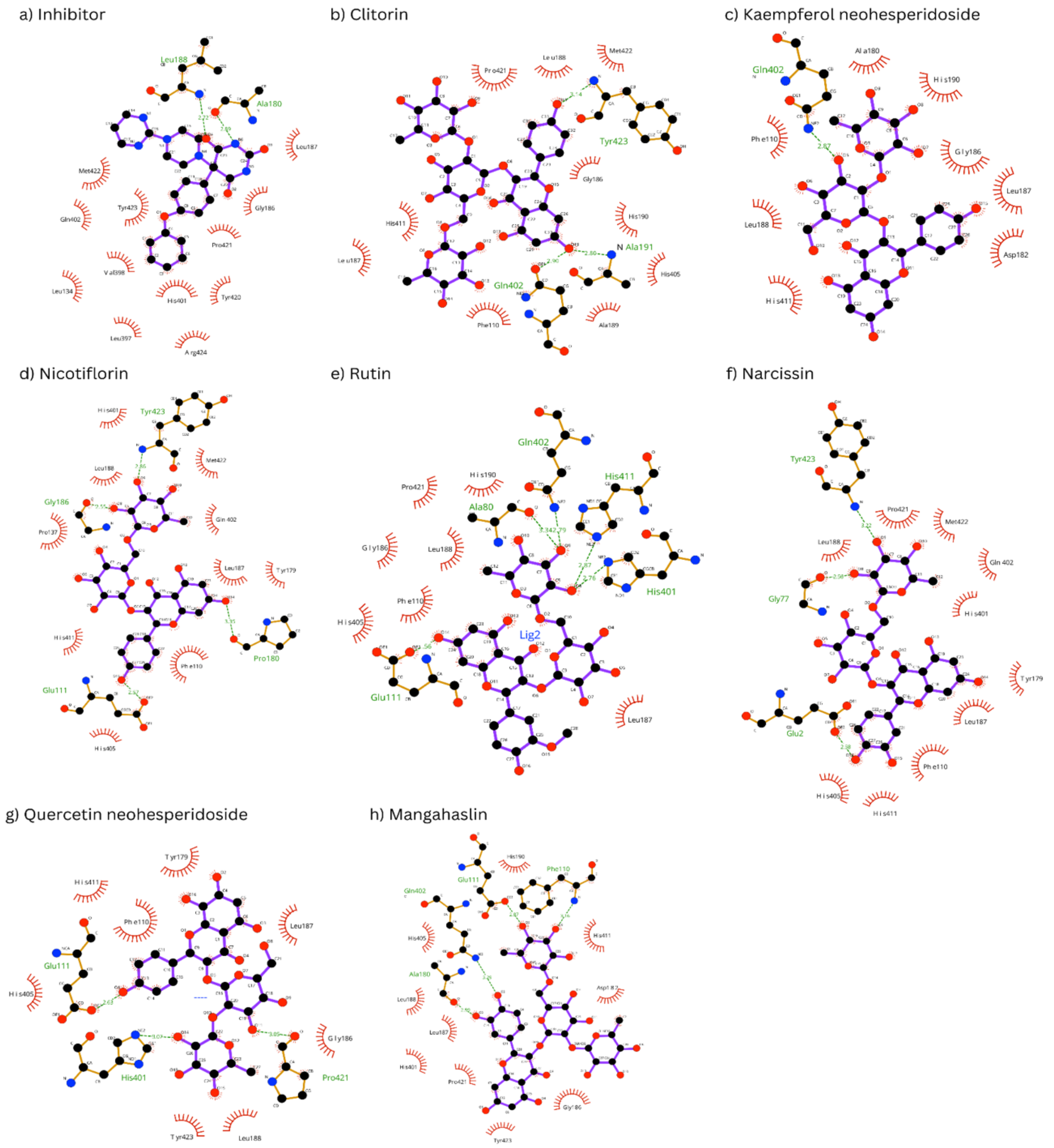

Supplementary Figure 3. Root Mean Square Deviation (RMSD) of the eight molecular dynamics simulations, in relation to the protein.

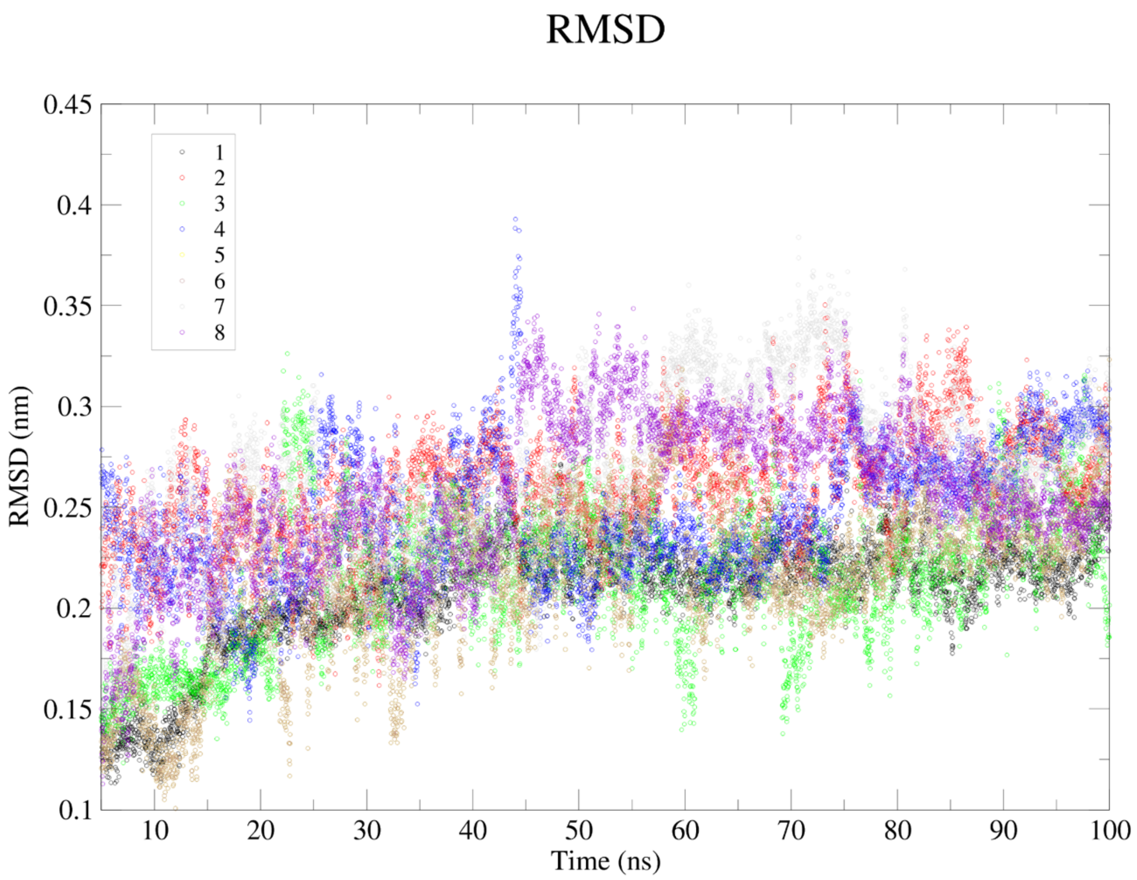

Supplementary Figure 6. Root Mean Square Fluctuation (RMSF) of the eight molecular dynamics simulations, in relation to the protein amino acids.

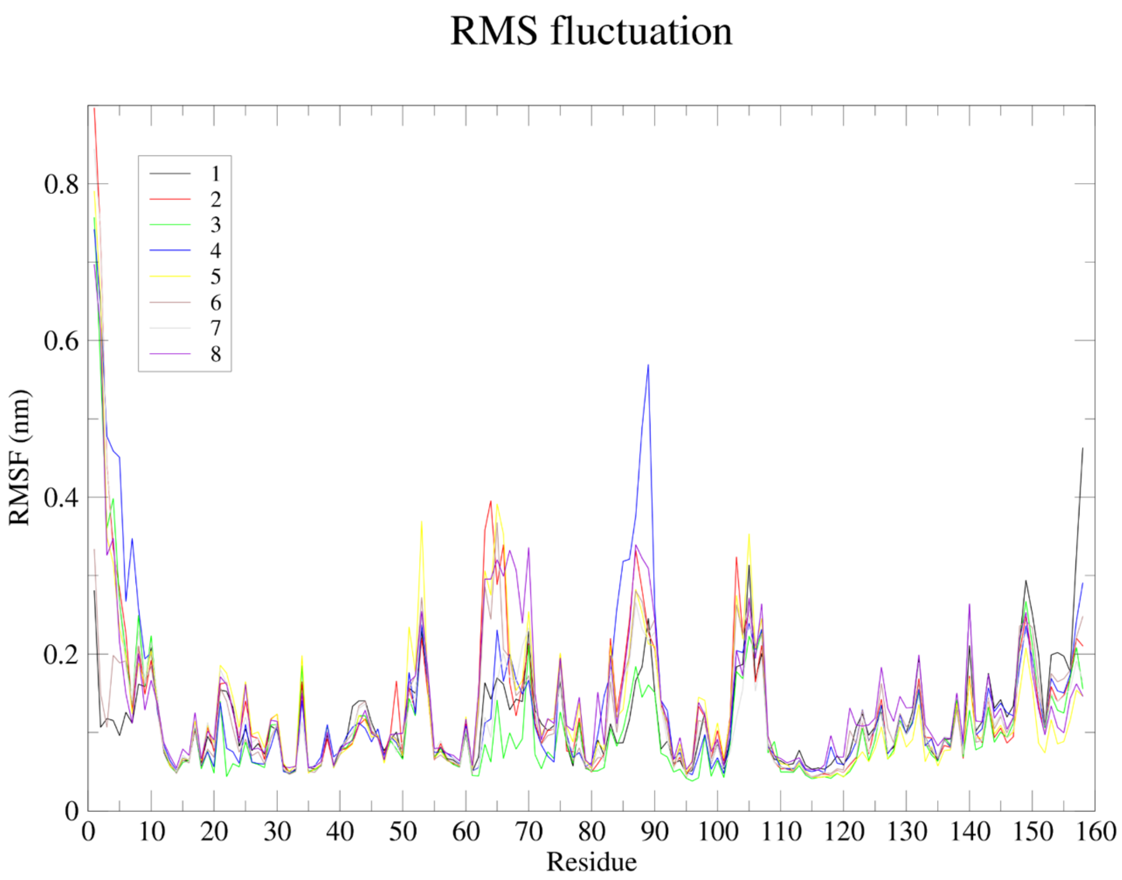

Supplementary Figure 7. Gelatinase activity in the supernatant from Raw264.7 macrophages stimulated with LPS and co-incubated with CC extract, tea, and the isolated flavonoids.

Extract and Tea

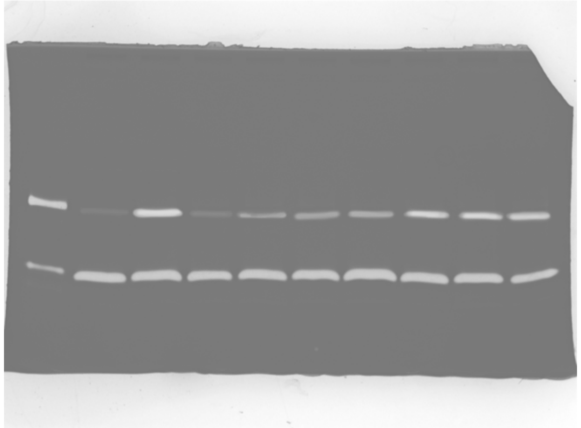

Manghaslin and Rutin.

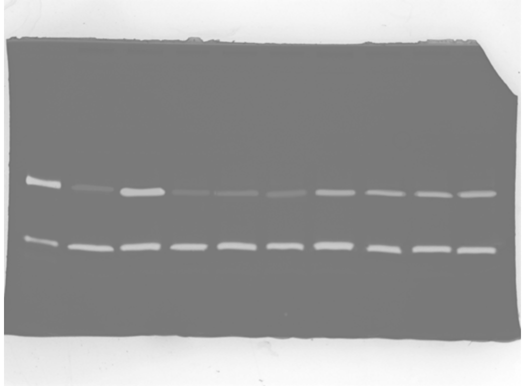

Q-neo and Clitorin

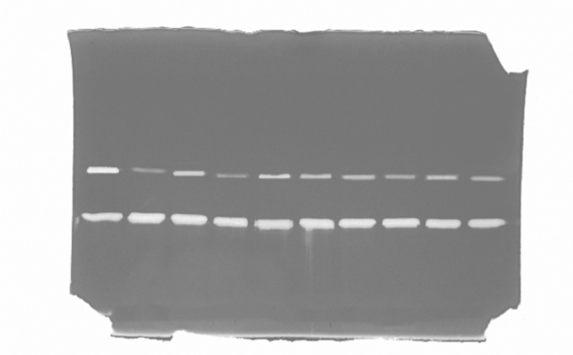

Nicotiflorin and K-neo

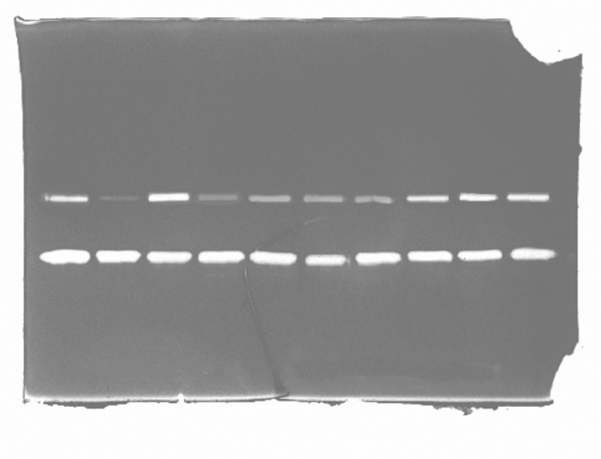

Narcissin

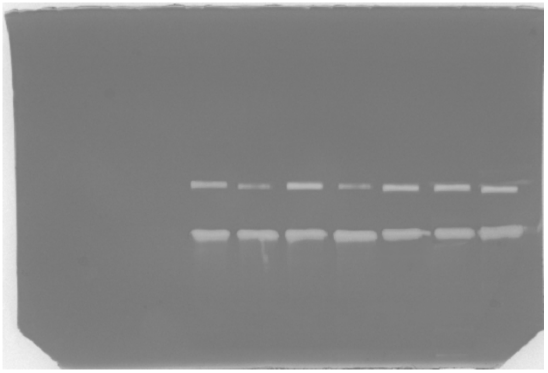

Supplement: Supplementary file 1 [file DataSheet1.PDF]
